# Anti-endoglin monoclonal antibody TRC105 prevents the increase of liver inflammatory biomarkers in a mouse model of cholestasis

**DOI:** 10.1007/s00018-026-06212-2

**Published:** 2026-04-29

**Authors:** Samira Eissazadeh, Ivana Nemeckova, Petra Fikrova, Nadia Alaei, Jana Urbankova Rathouska, Martina Vasinova, Seyedeh Niloufar Mohammadi, Katarina Tripska, Milos Hroch, Stanislav Micuda, Petr Nachtigal

**Affiliations:** 1 Department of Biological and Medical Sciences, Faculty of Pharmacy in Hradec Kralove, Charles University, Hradec Kralove, Czech Republic; 2 Department of Pediatric Hematology and Oncology, Second Faculty of Medicine, Charles University, Prague, Czech Republic; 3 Childhood Leukaemia Investigation Prague, Prague, Czech Republic; 4 Laboratory of Hemato-Oncology, Institute of Molecular Genetics, Czech Academy of Sciences, Prague, Czech Republic; 5 Department of Medical Biochemistry, Faculty of Medicine in Hradec Kralove, Charles University, Hradec Kralove, Czech Republic; 6 Department of Pharmacology, Faculty of Medicine in Hradec Kralove, Charles University, Hradec Kralove, Czech Republic; 7 Department of Biological and Medical Sciences, Faculty of Pharmacy in Hradec Kralove, Charles University, Hradec Kralove, Heyrovskeho, 1203, 500 05 Czech Republic

**Keywords:** Liver inflammatory biomarkers, Endoglin, Anti-endoglin antibody, Cholestasis

## Abstract

Cholestatic liver diseases are characterized by impaired bile flow, leading to inflammation and progression to cirrhosis and liver failure. Endoglin (ENG), a TGF-β co-receptor, regulates SMAD-dependent signalling and endothelial function. Carotuximab (TRC105) is an anti-ENG monoclonal antibody that binds to ENG. We hypothesized that treatment with TRC105 could prevent the upregulation of inflammatory biomarkers reflecting the activation of liver sinusoidal endothelial cells (LSECs) and hepatic inflammation during the progression of intrahepatic cholestasis (IC) in vivo by modulating the ENG and SMAD signaling pathways. IC was induced in male C57BL/6J mice by feeding with a 3,5-diethoxycarbonyl-1,4-dihydrocollidine (DDC) diet for 4 weeks. 18 mice were divided into three groups: a control group (chow diet), an IC group (DDC diet), and a rescue group (DDC diet + TRC105). The rescue group received TRC105 (15 mg/kg) twice weekly throughout the 4 weeks. We observed an inflammation in LSECs and the liver in IC animals characterized by overexpression of p65 NF-κB, ICAM-1, VCAM-1, endothelin-1, Galectin-3, CD11b, Ccl2, Ly6c, Il-1β, and Tnf-α, together with reduced expression of ENG and p-eNOS. TRC105 treatment prevented the downregulation of ENG and p-eNOS, the overexpression of inflammatory biomarkers, and the imbalance of SMAD signalling. We demonstrate for the first time that TRC105 prevents the upregulation of inflammatory biomarkers reflecting LSECs activation and hepatic inflammation, modulates ENG expression, and prevents the imbalance of the SMADs signalling during IC. These findings position ENG as a promising pharmacological target for alleviating inflammation in cholestatic liver disease, thereby opening a new avenue for translational research.

**Graphical abstract:**

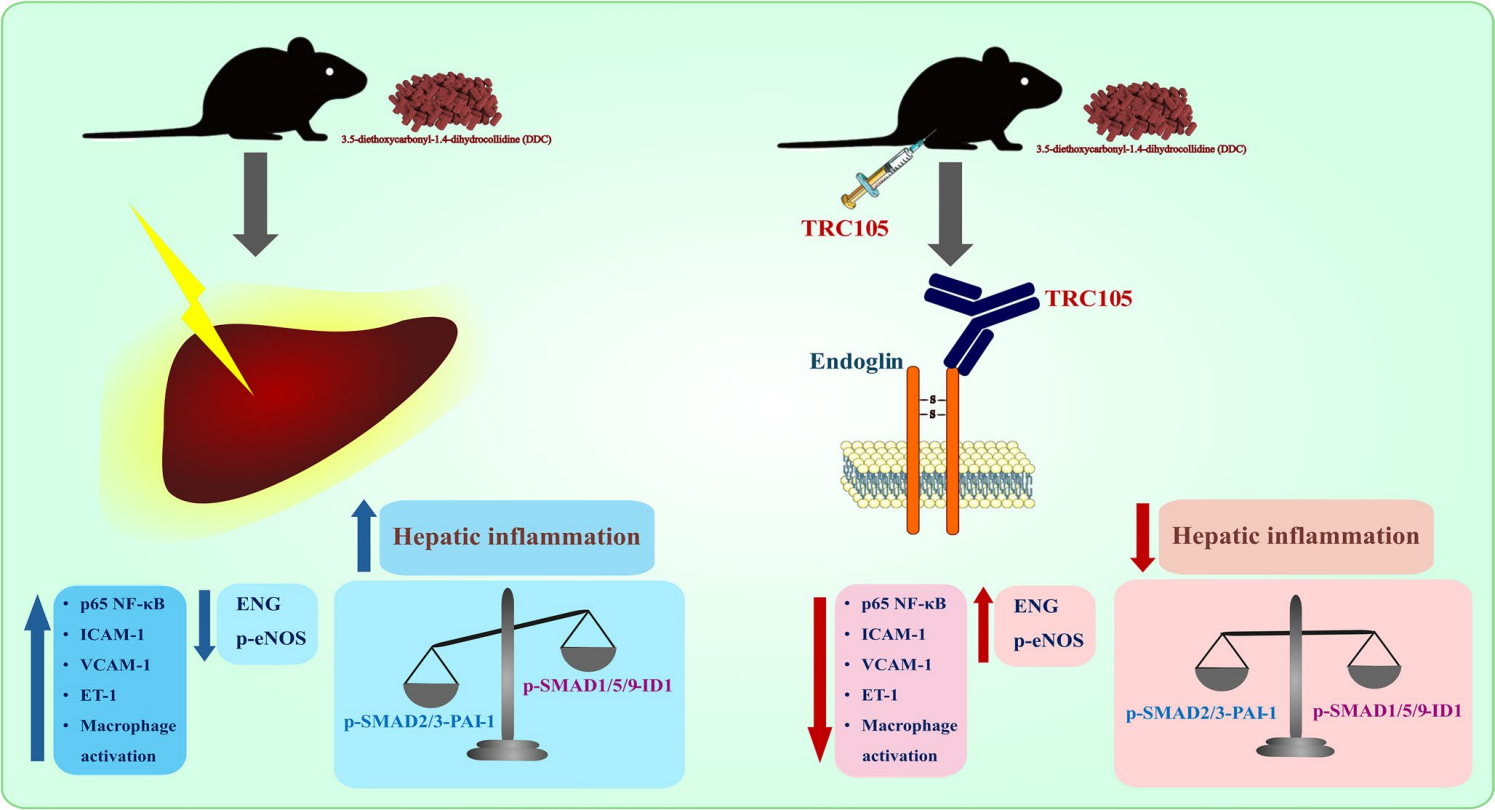

**Supplementary Information:**

The online version contains supplementary material available at 10.1007/s00018-026-06212-2.

## Introduction

In chronic intrahepatic cholestatic (IC) diseases such as primary biliary cholangitis, inflammation drives both the onset and progression of liver injury. Continuous damage to the bile ducts blocks bile flow, causing bile constituents, especially bile acids, to accumulate inside the liver. These retained bile acids trigger hepatocyte death, intensify the inflammatory cascade, and promote progressive fibrosis. They also encourage the growth of abnormal ductular cells that secrete pro-inflammatory and pro-fibrotic factors, amplifying hepatic damage even further [1, 2]. If left untreated, the combined inflammation and fibrosis advance to cirrhosis and ultimately end-stage liver disease [3]. Therefore, it is necessary to develop new therapeutic strategies for these cholangiopathies.

Cholestatic liver injury is closely associated with hepatic inflammation, in which nuclear factor kappa B (NF-κB) acts as a central regulator by upregulating pro-inflammatory cytokines such as tumour necrosis factor-alpha (TNF-α) and interleukin-1β (IL-1β). NF-κB activation also induces expression of adhesion molecules, intercellular adhesion molecule-1 (ICAM-1) and vascular cell adhesion molecule-1 (VCAM-1), facilitating immune cell infiltration and amplifying hepatic inflammation [4, 5]. Galectin-3, a β-galactoside-binding lectin, further promotes macrophage activation and NLR family pyrin domain-containing 3 (NLRP3) inflammasome signalling, contributing to cholestatic liver injury [6].

Endoglin (ENG) is a 180 kDa transmembrane glycoprotein considered a coreceptor for ligands of the transforming growth factor β (TGF-β) superfamily. There are two different ENG forms, namely membrane ENG and soluble ENG (sENG) [7].

Membrane ENG has been increasingly recognized for its role in liver pathology related to inflammation, liver sinusoidal endothelial dysfunction (LSED), and fibrosis, with some controversial outcomes. The membrane ENG is expressed mainly by endothelial cells [7] and liver sinusoidal endothelial cells (LSECs) [8], fibroblasts [9], hepatic stellate cells (HSCs) [10], activated monocytes, and macrophages [11], but not hepatocytes in the liver [12]. ENG promotes the TGF-β/ALK1 signalling, enhancing SMAD1/5 phosphorylation, which leads to the regulation of targets such as the inhibitor of DNA binding 1 (ID1) [13]. Interestingly, this ENG/SMAD1/5 axis regulates inflammation and supports vascular repair, whereas its dysregulation may trigger inflammatory responses that promote fibrotic remodelling [14–17]. Conversely, TGF-β-induced SMAD2/3/plasminogen activator inhibitor-1 (PAI-1) signalling primarily drives fibrosis and secondarily amplifies inflammation through extracellular matrix (ECM) deposition and immune cell recruitment [18–21].

Our previous research highlighted ENG’s active role in liver pathology. On the one hand, ENG has been reported to be increased and involved in metabolic dysfunction-associated steatohepatitis (MASH)-related LSED and fibrosis [8, 22]. On the other hand, reduced ENG expression was observed in cholestatic liver diseases simultaneously with proinflammatory activation of LSECs [8, 23]. Interestingly, although ENG is downregulated in cholestatic conditions, elevated serum concentrations of sENG, generated by matrix metalloproteinase-14 (MMP-14)-mediated cleavage, can be detected [8]. Indeed, sENG promotes a pro-inflammatory phenotype in endothelial cells, activating pathways such as NF-κB and increasing the expression of cytokines like IL-6 and TNF-α [24, 25]. However, the impact of modulation of ENG expression and signaling, especially in cholestatic liver disease, is yet unknown.

Carotuximab (TRC105, chimeric human IgG1) is a human therapeutic monoclonal antibody (mAb) that can bind to human ENG and has been studied in several clinical trials in oncology and age-related macular degeneration [26–29]. By binding to ENG, TRC105 disrupts BMP-9/ALK1-mediated signalling and enhances MMP-14-mediated cleavage of ENG, resulting in elevated serum concentrations of sENG [30].

Building on this established model and our prior findings on ENG function, we extended our investigation to evaluate the therapeutic potential of anti-ENG mAb strategies. Our previous findings highlighted the critical role of maintaining physiological ENG expression for the proper function of LSECs in vivo [8]. In continuation of this work, we further demonstrated that treatment with an anti-ENG mAb can prevent LSECs inflammation and fibrosis in a MASH animal model and reduce inflammatory responses in LSECs in vitro [22]. These results suggested that ENG may serve as a promising pharmacological target for addressing liver inflammation.

Therefore, the current study hypothesizes that treatment with the anti-ENG mAb TRC105 may inhibit the upregulation of inflammatory biomarkers reflecting LSECs activation and hepatic inflammation during the progression of IC in vivo by modulating the ENG and SMADs signalling cascades, representing the first investigation of anti-ENG mAb therapy in IC and providing new therapeutic insights for its treatment.

## Methods and materials

### Animals and diets

Male C57BL/6J mice, aged 9–10 weeks, were obtained from Velaz Company (Prague, Czech Republic). Following a two-week acclimation period, the animals were housed under controlled environmental conditions (22 ± 1 °C, 12-hour light/dark cycle, constant humidity). Throughout the study, the mice had *ad libitum* access to water and a standard rodent diet.

IC was induced using a 3,5-diethoxycarbonyl-1,4-dihydrocollidine (DDC) diet (NDC112-044, Ssniff, Germany), which has been widely used to induce a rapid onset of IC in mice, mimicking key features of the human disease [31], while control mice were fed standard chow pellets (1314–10 mm, Altromin, Germany). Eighteen mice were randomly assigned to three groups (*n* = 6 per group): [1] Control group, fed chow diet (Control); [2] IC group, fed DDC diet (DDC); and [3] Rescue treatment group, fed DDC diet and treated with TRC105, an anti-ENG mAb (DDC + TRC105).

During the 4-week feeding period, both DDC-fed groups received intraperitoneal injections twice weekly with either physiological saline (15 mg/kg body weight) or TRC105 (15 mg/kg body weight; TRACON Pharmaceuticals Inc., San Diego, CA, USA). The sample size was determined by power analysis using G*Power software, based on a non-parametric Mann–Whitney test with α = 0.05 to ensure sufficient statistical power. In accordance with the 3Rs principles and the Five Freedoms framework for animal welfare [32], the number of animals was reduced to six per group. This reduction also accounts for the anticipated variability in liver injury severity and the increased risk of mortality associated with DDC-induced hepatotoxicity and the administration of physiological saline or TRC105.

At the study endpoint (after the 4-week period), mice were euthanized by intraperitoneal administration of an overdose of ketamine (100 mg/kg) and xylazine (10 mg/kg). Blood was collected from the *inferior vena cava*, and liver tissues were harvested for subsequent analyses.

All animal procedures were approved by the Ethical Committee of Charles University, Faculty of Pharmacy in Hradec Kralove (Project No. MSMT-5793/2021–2, approval date: 4 May 2021), and were conducted in accordance with Czech Law No. 246/1992 Sb. The experimental protocol was also registered and approved by the International Register of Preclinical Trial Protocols (registration number PCTE0000622). All procedures complied with institutional and national guidelines for the care and use of laboratory animals and were reported in accordance with the ARRIVE guidelines to ensure transparency and reproducibility.

### Biochemical analysis

A 100 µL sample of fresh blood was applied to a Preventive Care Profile Plus cartridge (Abaxis, Griesheim, Hesse, Germany) to measure liver enzymes and total bilirubin. Analyses were performed using the VetScan2 analyser (Abaxis, Griesheim, Hesse, Germany).

### Quantitative real-time RT-PCR

Quantitative reverse transcription-polymerase chain reaction (qRT-PCR) was performed using the QuantStudio 6 Flex Real-Time PCR System (Applied Biosystems, Thermo Fisher Scientific, MA, USA) as previously described [33]. Gene expression levels were normalized to Glyceraldehyde 3-phosphate dehydrogenase (*Gapdh*) as a reference gene. The primer sequences used in this analysis are listed in Supplementary Table 1.

### Western blot analysis

Western blot analysis of total and membrane liver fractions was performed as previously described [22], with minor modifications. Briefly, 25 µg of protein per sample was loaded and separated by SDS-PAGE on 6%, 8%, 10%, or 12% polyacrylamide gels, selected according to the molecular weight of the target proteins, followed by transfer onto nitrocellulose membranes (Bio-Rad Laboratories, Hercules, CA, USA). Membranes were blocked for 1 to 1.5 h in 5% non-fat dry milk (Bio-Rad Laboratories) prepared in Tris-buffered saline containing 0.1% Tween-20 (TBS-T; Millipore Sigma, Burlington, MA, USA). Primary antibodies were incubated overnight at 4 °C at optimized concentrations (see Supplementary Table 2). After six washes with TBS-T, membranes were incubated for 1 to 1.5 h at room temperature on a shaker with appropriate HRP-conjugated secondary antibodies (see Supplementary Table 2). Following further washes, protein bands were visualized using the ChemiDoc™ MP Imaging System (Bio-Rad Laboratories), and band intensities were quantified with Image Lab software version 6.1 (Bio-Rad Laboratories). Brightness and contrast adjustments were applied uniformly across the entire image and did not alter the original data. Protein expression levels were normalized to GAPDH.

### Comparative analysis of p-SMAD2/3 and p-SMAD1/5 signalling pathways during DDC feeding and TRC105 treatment

To investigate potential shifts between the p-SMAD2/3 and p-SMAD1/5 signalling pathways during IC induced by the DDC diet and TRC105 treatment in mice, we conducted a comparative analysis of phosphorylated SMAD proteins. Levels of p-SMAD2/3 and p-SMAD1/5 were measured in liver samples from all three experimental groups: Control, DDC, and DDC+TRC105. The aim was to determine the ratio of p-SMAD2/3 to p-SMAD1/5 under healthy conditions, during IC, and following TRC105 treatment, and to compare these ratios across groups to identify potential shifts in pathway activation.

Western blot analysis was performed to evaluate the activation of the TGF-β pathway (via SMAD2/3) and the BMP9 pathway (via SMAD1/5), with signal intensities quantified to assess the relative activation of these pathways. For each group, two separate membranes were prepared: one probed for p-SMAD2/3 and the other for p-SMAD1/5. To ensure consistent exposure and accurate comparison, membranes from each group were imaged simultaneously using identical settings on the ChemiDoc imaging system. The experiment was independently replicated three times using biologically distinct samples, with six samples per group, to confirm reproducibility and enable statistical analysis. The ratio of p-SMAD2/3 to p-SMAD1/5 was calculated for each group and compared between groups to detect any shifts in SMADs signalling.

This comparative ratio-based analysis was developed in our study to assess changes in SMADs signalling balance in IC and following treatment with TRC105.

### sENG protein analysis

sENG serum concentrations were semi-quantitatively measured using a Western blot-based method. To ensure accurate detection, plasma samples were concentrated prior to analysis to increase protein content, as described previously [34]. Although ELISA is the standard method for sENG quantification, Western blotting was employed in this study because interference of the ENG-blocking mAb TRC105 with the ELISA detection antibody would have resulted in inaccurate measurements. Western blotting, being less susceptible to such Ab interference, allowed semi-quantitative assessment of sENG serum concentrations with higher specificity.

### Histology and immunohistochemical analysis

Liver tissues were fixed in 4% paraformaldehyde and subsequently embedded in paraffin. Serial cross-Sect. (4.5 μm thick) were prepared and stained using the Sirius Red/Fast Green Collagen Staining Kit (9046, Chondrex, WA, USA) for histological evaluation. For immunohistochemical (IHC) analysis, additional 4.5 μm-thick sections were cut using a microtome and mounted on gelatin-coated slides. Four animals per experimental group were analyzed, with five sections per animal. Slides were incubated with the respective primary antibody (Supplementary Table 3). Detection was performed using a biotinylated secondary antibody, followed by the horseradish peroxidase-labeled avidin-biotin complex (ABC kit PK-4001, Vector Laboratories, CA, USA). Signal development was achieved with the ImmPACT^®^ VIP Substrate Kit (SK-4605, Vector Laboratories, CA, USA), with hematoxylin counterstaining. The specificity of staining was confirmed using isotype-matched control antibodies. Images were captured using an Olympus AX70 microscope equipped with a Nikon DS-Fi3 high-definition color camera and analyzed with NIS image analysis software (Laboratory Imaging, Czech Republic).

### Statistical analysis

Statistical analyses were carried out using GraphPad Prism version 10 (GraphPad Software Inc., San Diego, CA, USA). Data are presented as median values with interquartile ranges. Comparisons between groups were performed using the non-parametric Mann-Whitney test, except for the analysis shown in Fig. 7A, where one-way ANOVA was additionally applied. A *p-value* of less than 0.05 was considered statistically significant.

## Results

### TRC105 treatment prevents liver weight and liver-to-weight ratios from increasing, but not liver enzymes, in DDC-fed mice.

**Fig. 1:**
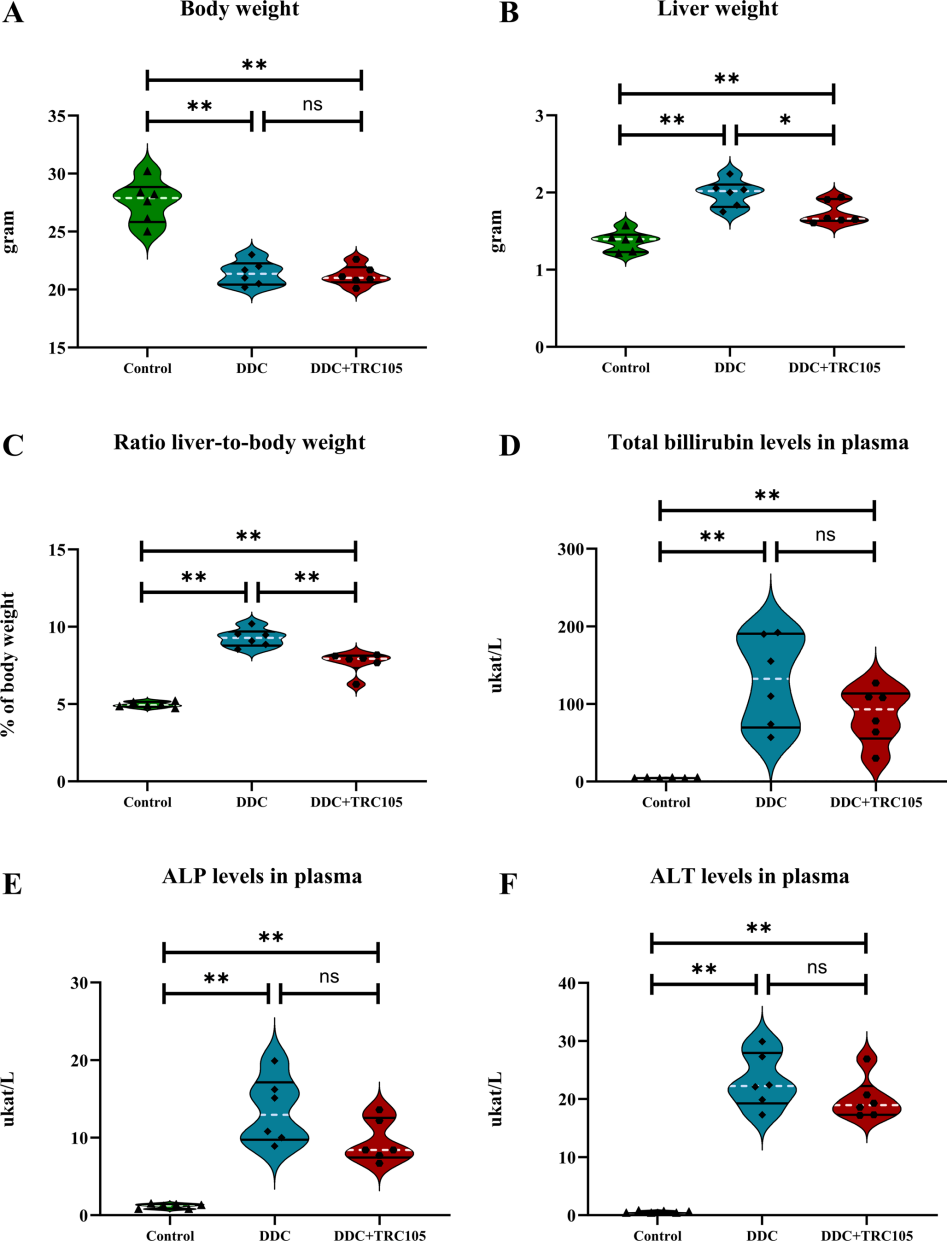
Effect of TRC105 on liver damage parameters in a mouse model of IC. Body weight (**A**), liver weight (**B**), and liver-to-body weight ratio (**C**). The levels of total bilirubin (**D**), liver enzymes, ALP (**E**), and ALT (**F**) in plasma. Data are presented as median with interquartile range. Statistical analysis was performed using the Mann-Whitney test, ns (not significant), * *p* < 0.05, ** *p* < 0.01; *n* = 6 animals per group

In DDC-fed mice, body weight was significantly decreased compared to the control group (Fig. 1A). In contrast, liver weight (Fig. 1B) and the liver-to-body weight ratio (Fig. 1C) were markedly increased in the DDC-fed groups, indicating hepatomegaly and hepatic impairment. Although TRC105 treatment for 4 weeks did not significantly affect body weight in DDC mice (Fig. 1A), it significantly prevented both liver weight (Fig. 1B) and the liver-to-body weight ratio increase when compared to the DDC group (Fig. 1C). Levels of total bilirubin (Fig. 1D), alkaline phosphatase (ALP) (Fig. 1E), and alanine transaminase (ALT) (Fig. 1F) were significantly elevated in the plasma of DDC-fed mice. However, TRC105 treatment did not result in a statistically significant change in these parameter levels compared to the DDC group. These results suggest that TRC105 may exert a beneficial effect on liver hepatomegaly, although its influence on liver injury markers was limited, possibly due to the relatively short 4-week treatment duration.

### TRC105 does not prevent fibrosis progression in DDC-fed mice

**Fig. 2:**
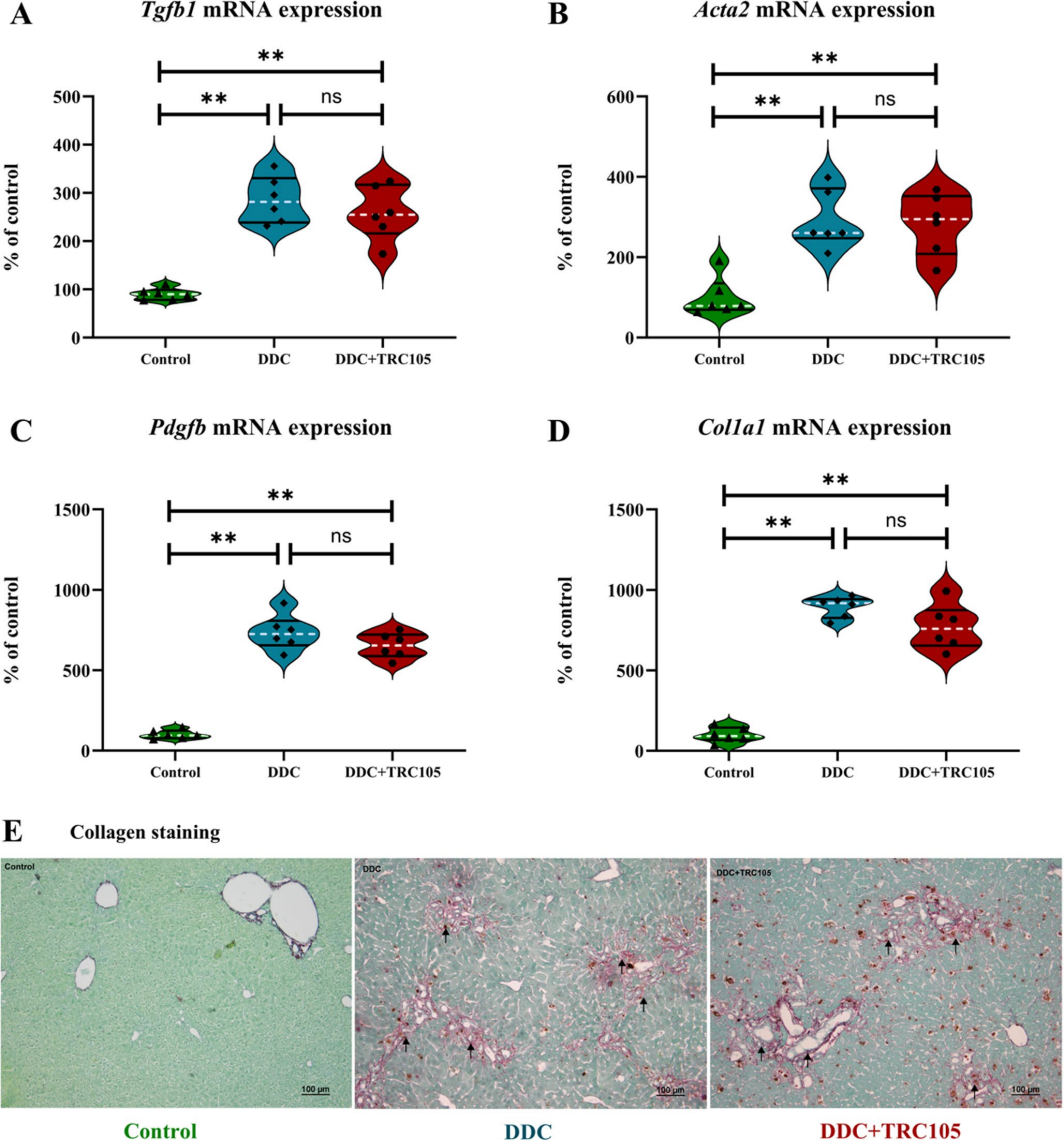
Effect of TRC105 treatment on hepatic fibrogenic gene expression in an IC mouse model. Relative mRNA expressions of *Tgfb1* (**A**), *Acta2* (**B**), *Pdgfb* (**C**), and *Col1a1* (**D**). Collagen (arrows) stained by Sirius Red/Fast Green in liver tissues (E). Scale bar 100 μm, 100× magnification. Data are presented as median with interquartile range. Statistical analysis was performed using the Mann-Whitney test, ns (not significant), ** *p* < 0.01; *n* = 6 animals per group

Significant increases in the mRNA expressions of *Tgfb1* (Fig. 2A), actin alpha 2 (*Acta2*) (Fig. 2B), platelet-derived growth factor subunit B (*Pdgfb*) (Fig. 2C), and collagen type I alpha 1 chain (*Col1a1*) (Fig. 2D) were observed in DDC-fed mice compared to the control group. However, TRC105 treatment did not significantly affect the expression of any of these fibrosis-related genes, indicating that TRC105 is ineffective in inhibiting fibrosis progression in this mouse model of IC. Sirius Red/Fast Green staining for the detection of collagen showed a reaction only in the wall of the central vein, arterioles, and venules in the portal area in the control mice (Fig. 2E). DDC feeding markedly increased Sirius Red/Fast Green collagen deposition in portal triads, consistent with advanced fibrogenesis and a robust ductular reaction characterized by ductular proliferation and cholangitis, however, without no impact of TRC105 treatment (Fig. 2E).

### TRC105 treatment modulates ENG expression, and the balance of SMAD1/5 and SMAD2/3 signalling cascades in DDC-fed mice

**Fig. 3:**
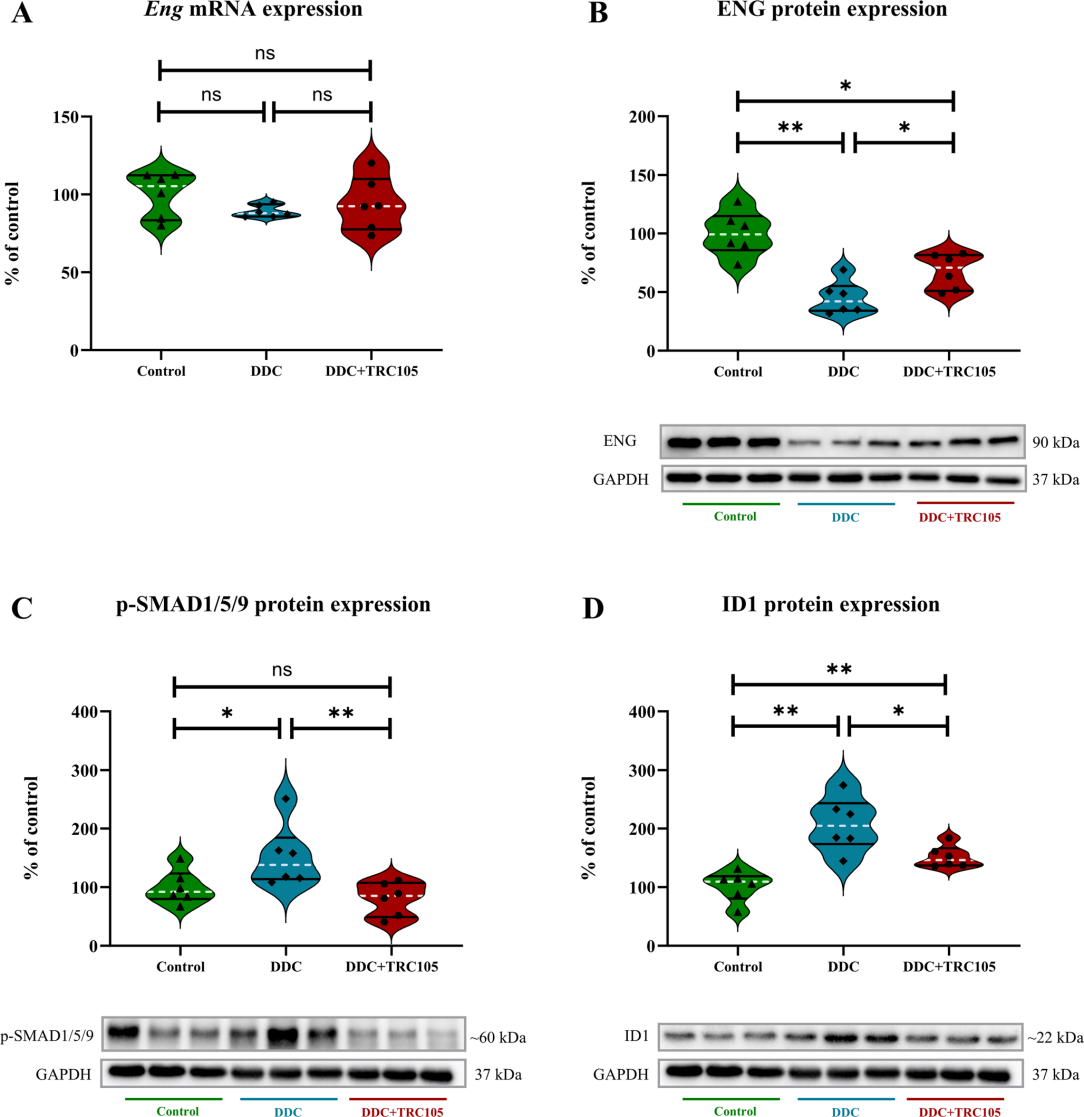

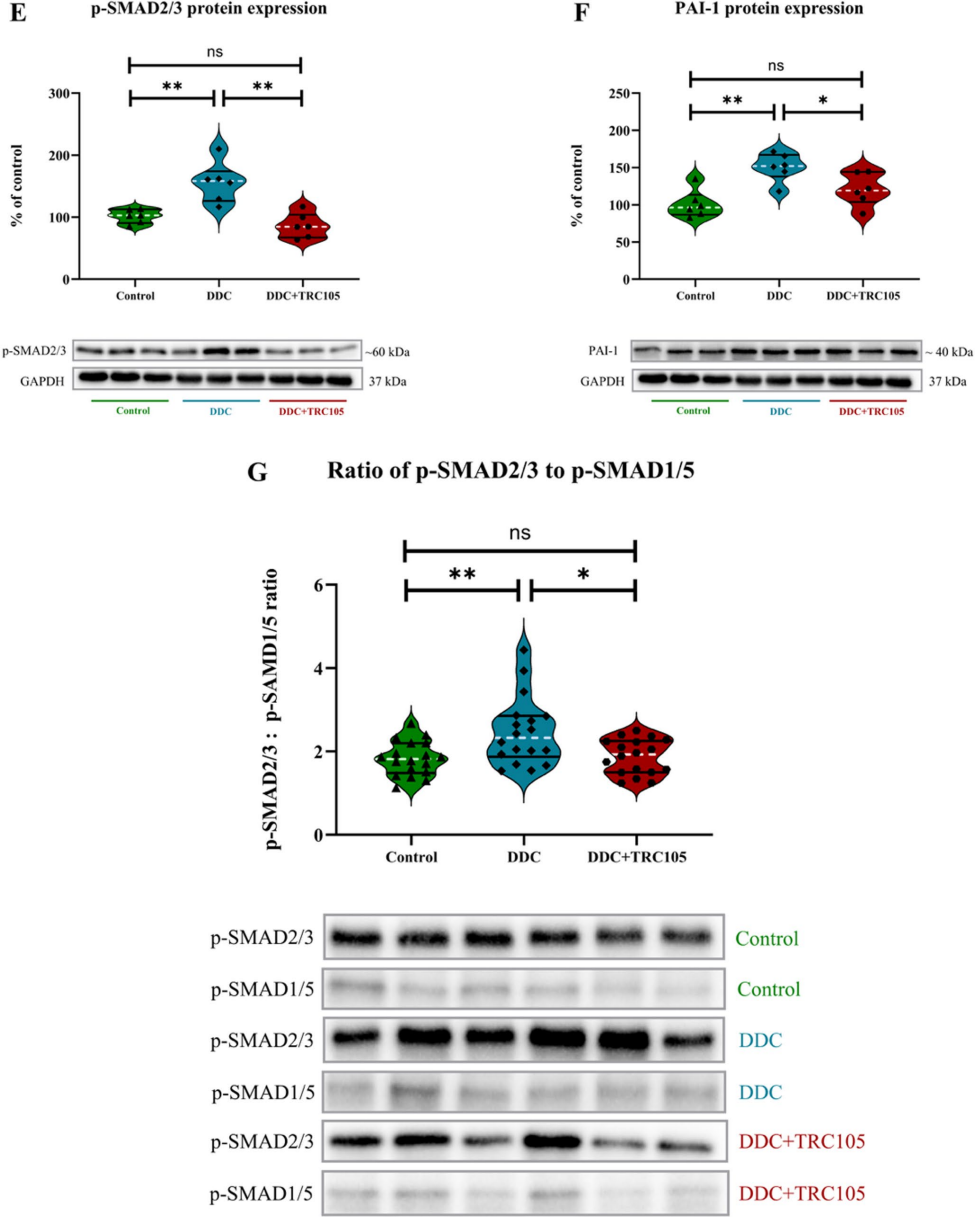
Effect of TRC105 on ENG expression and the signaling balance between SMAD1/5 and SMAD2/3 pathways in the DDC animal model. Relative *Eng* mRNA expression (**A**), protein expression levels of ENG (**B**), p-SMAD1/5/9 (**C**), ID1 (**D**), p-SMAD2/3 (**E**), and PAI-1 (**F**). The ratio of p-SMAD2/3 to p-SMAD1/5/9 (**G**) is shown. Data are presented as median with interquartile range. Statistical analysis was performed using the Mann-Whitney test, ns (not significant), * *p* < 0.05, ** *p* < 0.01; *n* = 6 animals per group. For each protein (panels **B**-**F**), two gels were run to include all samples from the three groups. Both gels were processed in parallel under identical conditions. Automatic exposure settings were applied uniformly across all blots. Blots were cropped solely to fit the figure layout, without removing or rearranging any lanes or samples. Full, uncropped blots are provided in the supplementary Western blot file. Image processing, including brightness and contrast adjustments, was performed uniformly across the entire image using Image Lab software version 6.1 (Bio-Rad Laboratories) and did not alter the original data

Although ENG mRNA expression (Fig. 3A) did not show significant changes in DDC-fed mice, its protein expression was significantly lower in the DDC group (Fig. 3B) compared to the control group. At the same time, TRC105 treatment significantly prevented the decrease of ENG protein expression (Fig. 3B) compared to DDC animals.

Moreover, protein expressions of p-SMAD1/5/9 (Fig. 3C), ID1 (Fig. 3D), p-SMAD2/3 (Fig. 3E), and PAI-1 (Fig. 3F) were significantly increased in the DDC group compared to the control mice, indicating activation of the p-SMAD1/5/9-ID1 pathway as well as the p-SMAD2/3-PAI-1 pathway. However, TRC105 effectively prevented the elevation of all these proteins compared to DDC-fed mice, suggesting that anti-ENG mAb modulates both signalling pathways.

The ratio of p-SMAD2/3 to p-SMAD1/5/9 protein expression was significantly increased in DDC-fed mice by approximately 26% compared to the control group (Fig. 3G), indicating a shift toward the p-SMAD2/3 signalling cascade and a predominance of this pathway during IC. In contrast, this ratio was reduced by approximately 24% in TRC105-treated mice compared to DDC animals (Fig. 3G), revealing TRC105’s potential to rebalance the SMADs pathway activity.

### TRC105 treatment increases sENG serum concentrations via enhanced MMP-14-mediated shedding

**Fig. 4:**
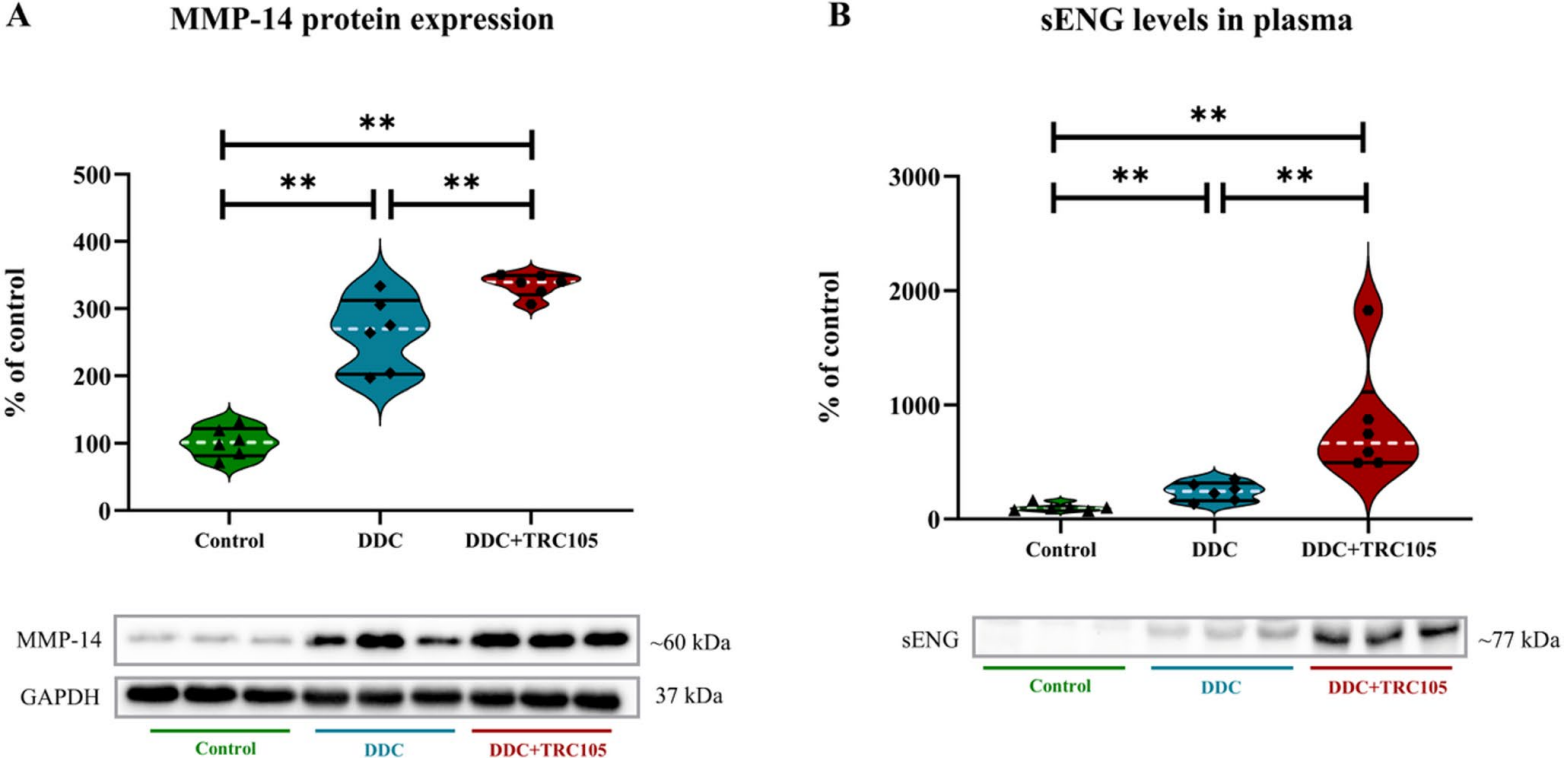
Anti-ENG mAb TRC105 enhances sENG release by increasing the shedding of membrane-bound ENG. Protein expression of MMP-14 (**A**) and sENG serum concentrations (**B**) are shown. Data are presented as median with interquartile range. Statistical analysis was performed using the Mann-Whitney test, **p < 0.01; *n* = 6 animals per group. For each protein, two gels were run to include all samples from the three groups. Both gels were processed in parallel under identical conditions. Automatic exposure settings were applied uniformly across all blots. Blots were cropped solely to fit the figure layout, without removing or rearranging any lanes or samples. Full, uncropped blots are provided in the supplementary Western blot file. Image processing, including brightness and contrast adjustments, was performed uniformly across the entire image using Image Lab software version 6.1 (Bio-Rad Laboratories) and did not alter the original data

MMP-14 protein expression was significantly increased in DDC-fed mice compared to the control group (Fig. 4A). Remarkably, this upregulation persisted and was further enhanced following TRC105 treatment, resulting in a significant increase in MMP-14 levels in TRC105-injected mice (Fig. 4A).

In parallel, sENG concentration was significantly elevated in the serum of DDC-fed mice compared to controls, and TRC105 treatment led to an even greater increase (Fig. 4B), suggesting that TRC105 may increase the shedding of membrane-bound ENG, potentially mediated by MMP-14.

### TRC105 treatment prevents an increase in biomarkers of inflammation in LSECs and the liver in DDC-fed mice

**Fig. 5:**
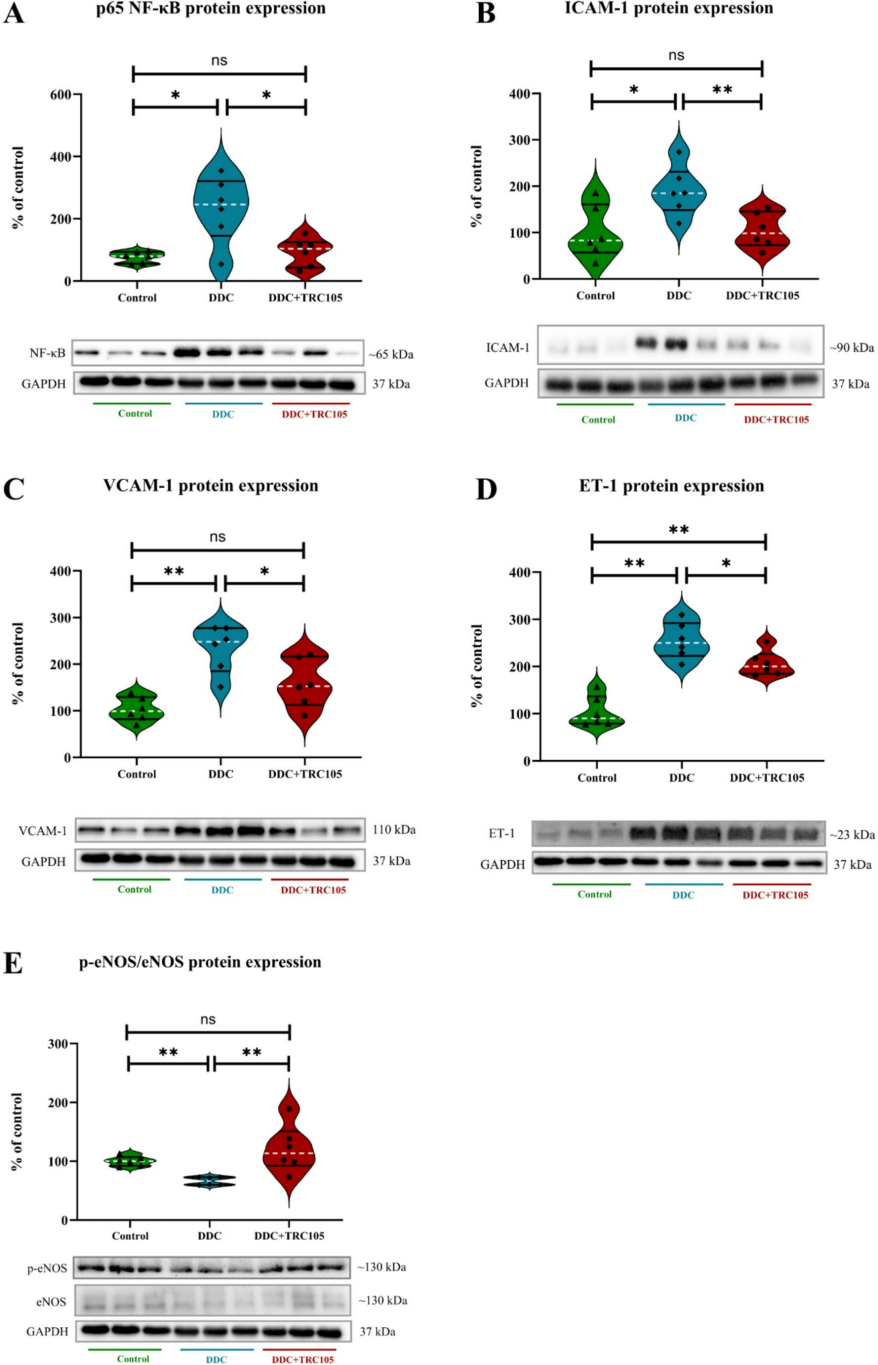
TRC105 treatment effects on biomarkers of LSECs and liver inflammation in a mouse model of IC. Protein expressions of p65 NF-κB (**A**), ICAM-1 (**B**), VCAM-1 (**C**), ET-1 (**D**), and p-eNOS/eNOS ratio (**E**) are shown. Statistical analysis was performed using the Mann-Whitney test, ns (not significant), * *p* < 0.05, ** *p* < 0.01; *n* = 6 animals per group. For each protein, two gels were run to include all samples from the three groups. Both gels were processed in parallel under identical conditions. Automatic exposure settings were applied uniformly across all blots. Blots were cropped solely to fit the figure layout, without removing or rearranging any lanes or samples. Full, uncropped blots are provided in the supplementary Western blot file. Image processing, including brightness and contrast adjustments, was performed uniformly across the entire image using Image Lab software version 6.1 (Bio-Rad Laboratories) and did not alter the original data

Protein expression levels of p65 NF-κB, ICAM-1, and VCAM-1 were significantly upregulated in DDC mice compared to the control group (Fig. 5A-C), reflecting endothelial activation and inflammation in the liver. Treatment with TRC105 significantly prevented the increase in expression of all three markers (Fig. 5A-C), efficiently reversing the inflammatory response induced by DDC feeding. These results indicated that TRC105 suppresses the activation of these inflammatory biomarkers in this mouse model of IC.

Endothelin-1 (ET-1) protein expression was significantly upregulated in DDC-fed mice compared to control animals (Fig. 5D), reflecting enhanced vasoconstrictive and pro-inflammatory signalling associated with endothelial activation. TRC105 treatment significantly prevented the increase in ET-1 protein expression (Fig. 5D).

Conversely, phosphorylated endothelial nitric oxide synthase (p-eNOS) was significantly downregulated in the DDC group (Fig. 5E), while TRC105 prevented p-eNOS expression reduction compared to DDC-fed mice (Fig. 5E), implying a potential recovery of eNOS expression and improved endothelial homeostasis.

### TRC105 treatment attenuates hepatic macrophage activation and infiltration in the liver of cholestatic mice

**Fig. 6:**
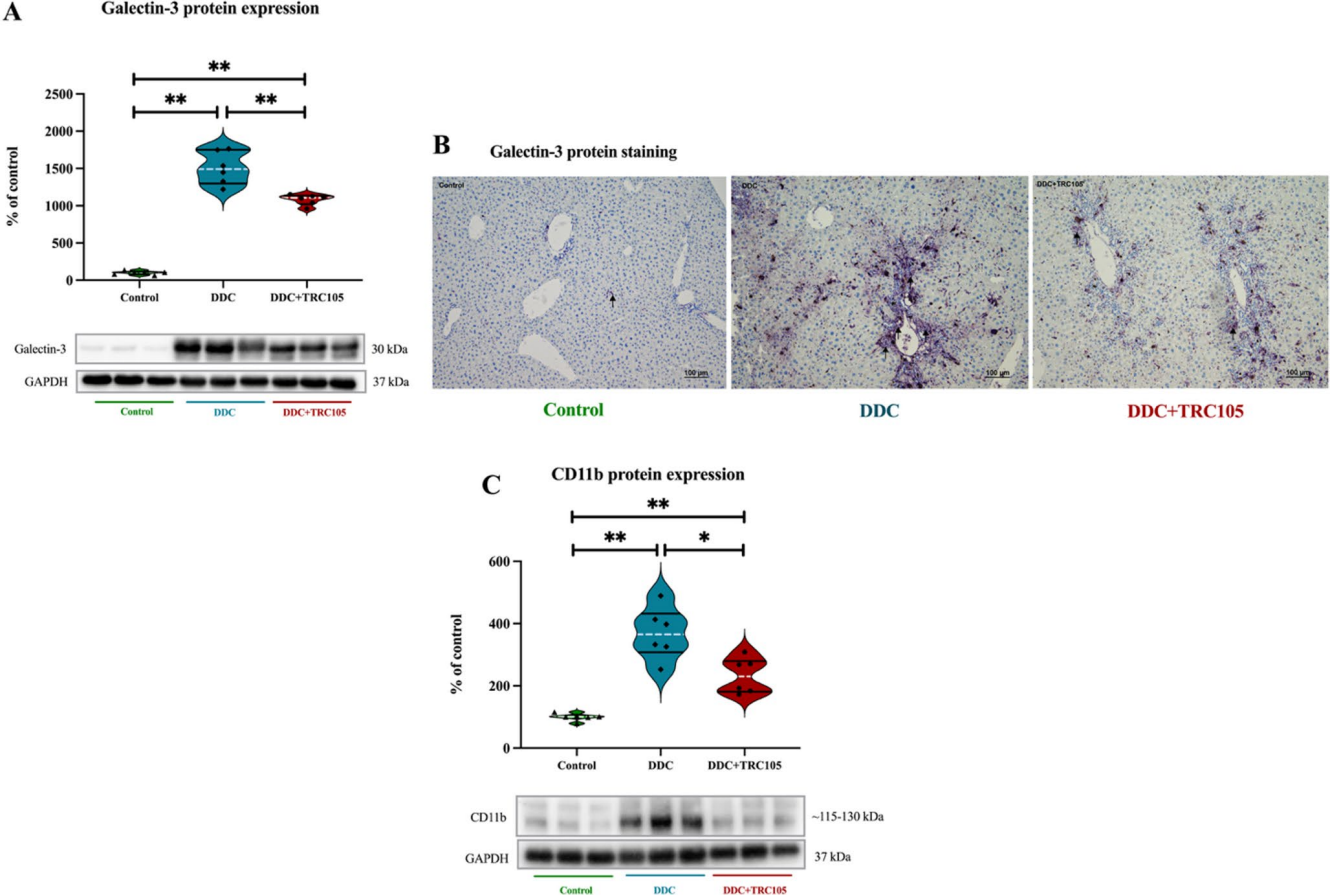
TRC105 treatment reduces hepatic macrophage activation and infiltration in cholestatic mouse livers. Protein expressions of Galectin-3 (**A**) and CD11b (**C**), are shown. Localization of Galectin-3 in Control, DDC, and DDC+TRC105 mice (arrows) (**B**). Scale bar 100 μm, 100× magnification. Data are presented as median with interquartile range. Statistical analysis was performed using the Mann-Whitney test, ns (not significant), * *p* < 0.05, ** *p* < 0.01; *n* = 6 animals per group. For each protein, two gels were run to include all samples from the three groups. Both gels were processed in parallel under identical conditions. Automatic exposure settings were applied uniformly across all blots. Blots were cropped solely to fit the figure layout, without removing or rearranging any lanes or samples. Full, uncropped blots are provided in the supplementary Western blot file. Image processing, including brightness and contrast adjustments, was performed uniformly across the entire image using Image Lab software version 6.1 (Bio-Rad Laboratories) and did not alter the original data

Galectin-3 protein expression, a marker commonly associated with activated macrophages, was significantly increased in DDC-fed mice compared to control animals (Fig. 6A), indicating enhanced macrophage-mediated inflammation in IC. In the DDC diet-fed animals, Galectin-3 positivity was highly increased in portal areas, showing massive inflammatory cell infiltration in these areas (Fig. 6B). In addition, TRC105 treatment significantly prevented the increase of Galectin-3 protein expression and macrophage infiltration (Fig. 6A-B), suggesting that TRC105 decreases macrophage activation and associated inflammatory response in DDC-fed mice.

Protein expression of CD11b (Fig. 6C), a marker associated with activated and infiltrating macrophages, was markedly elevated in the DDC group compared with the control group, reflecting increased monocyte/macrophage recruitment in the cholestatic liver. TRC105 treatment significantly attenuated the DDC-induced upregulation of CD11b (Fig. 6C), consistent with the reduction in Galectin-3 expression (Fig. 6A-B), suggesting reduced macrophage activation and infiltration following TRC105 administration.

These findings suggest that TRC105 prevents macrophage activation and inflammatory infiltration in the cholestatic liver.

### TRC105 prevented the increase in liver inflammation in the cholestatic mice

**Fig. 7:**
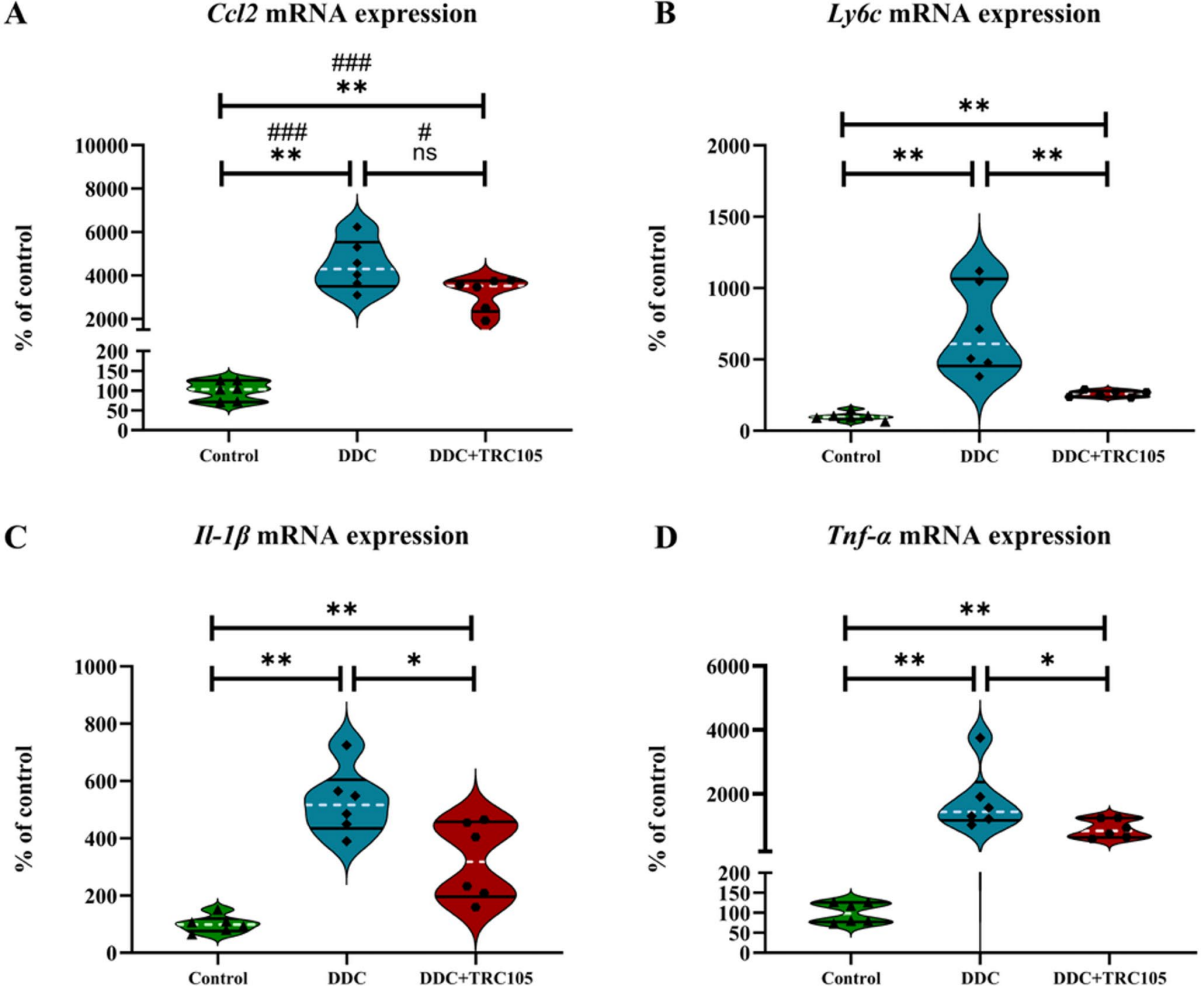
Impact of TRC105 treatment on hepatic proinflammatory gene expression in the IC mouse model. Relative mRNA expression of *Ccl2* (**A**), *Ly6c* (**B**), *Il-1β* (**C**), and *Tnf-α* (**D**). Data are presented as median with interquartile range. Statistical analysis was performed using the Mann–Whitney test for panels **A**–**D** and one-way ANOVA for panel **A** (*Ccl2*). ns, not significant; *p < 0.05; #*p* < 0.05; ** *p* < 0.01; ###*p* < 0.001; *n* = 6 animals per group

Hepatic mRNA expression levels of C-C motif chemokine ligand 2 (*Ccl2*) (Fig. 7A), Lymphocyte antigen 6 complex, locus C (*Ly6c*) (Fig. 7B), *Il-1β* (Fig. 7C), and *Tnf-α* (Fig. 7D), established proinflammatory markers [35], were significantly elevated in DDC-fed mice compared to the control group, confirming induction of liver inflammation in the IC model. Treatment with anti-ENG mAb TRC105 significantly inhibited the increase in these biomarkers (Fig. 7A–D). These results indicated that TRC105 suppresses the upregulation of liver inflammatory markers in the IC mouse model.

### Discussion

In this study, we provide the first evidence that treatment with TRC105, an anti-ENG mAb, significantly prevents the increase of inflammatory biomarkers reflecting LSECs activation and hepatic inflammation in a murine model of IC by modulating ENG expression and rebalancing the SMADs signalling pathway.

Interestingly, we previously demonstrated that ENG expression is altered by both IC and MASH, predominantly in LSECs. Thus, we proposed that maintaining physiological ENG protein levels in LSECs is essential for preserving endothelial function [8]. Consequently, our earlier study demonstrated that anti-ENG mAb prevented inflammation and fibrosis in LSECs in a MASH animal model and suppressed inflammatory responses in LSECs in vitro [22]. However, the specific effects of anti-ENG mAb on inflammation and fibrosis during the progression of IC remain to be elucidated.

Therefore, in this study, we hypothesized that blocking ENG with TRC105, an anti-ENG mAb, would prevent the progression of hepatic inflammation and activation of LSECs and fibrosis during the development of IC in vivo.

TRC105 treatment significantly reduced liver weight and the liver-to-body weight ratio in DDC-fed mice, indicating potential protection against hepatomegaly, a key feature of IC [36]. The observed reduction in liver weight and liver-to-body weight ratio is consistent with our previous finding [22] and may suggest that ENG and its signalling partially contributes to liver enlargement in the mouse model of IC.

TRC105 treatment did not significantly alter plasma ALT, AST, or total bilirubin levels in DDC-fed mice. As these markers reflect hepatocyte injury and cholestatic bile accumulation driven mainly by direct hepatotoxicity and bile duct obstruction in the DDC model [37], this likely suggests that ENG modulation does not directly normalize these biochemical parameters since ENG is not expressed by hepatocytes [12]. As expected, DDC feeding led to a significant upregulation of *Tgfb1*, *Acta2*, *Col1a1*, and *Pdgfb*, canonical markers of fibrogenesis, reflecting robust activation of pro-fibrotic pathways in response to the chronic induction of IC. These findings are consistent with previous reports demonstrating DDC-induced cholestasis, activation of HSCs, and ECM deposition [31, 35]. TRC105 treatment did not significantly prevent the increase in expression of any fibrosis-related genes. It has been demonstrated that during liver fibrosis, ENG is significantly upregulated in activated HSCs, the primary collagen-producing effector cells in the liver [38]. However, in this study, ENG blockade via TRC105 may be insufficient to affect fibrosis progression in this model of established IC liver injury. One possible explanation might be that ENG is not a dominant driver of TGF-β–mediated fibrogenesis in the DDC model, unlike in MASH [22], or that its inhibition requires a longer treatment duration.

Subsequently, we investigated the modulation of ENG expression and sENG serum concentration during IC and TRC105 treatment.

The DDC diet significantly decreased hepatic ENG expression while elevating sENG serum concentrations, consistent with our previous IC model results [8, 23]. In the IC model, ENG protein levels declined despite stable mRNA expression, suggesting post-transcriptional regulation. Post-transcriptional modulation and enhanced protein degradation are increasingly recognized as key contributors to altered protein expression in cholestatic liver disease, independent of transcriptional changes, a phenomenon supported by findings from various experimental models [39–42]. The ability of TRC105 to prevent the reduction in ENG protein expression may suggest a protective role in preserving ENG availability in endothelial cells, potentially through ENG–TRC105 complex formation, as TRC105 binds to a region that overlaps with the BMP9-binding site on ENG [43]. Since ENG is critical for endothelial function and vascular integrity this effect may help preserve both endothelial health and vascular homeostasis in the IC liver microenvironment [44, 45].

In addition, our results indicate that TRC105 significantly influences sENG shedding in the DDC-induced mouse model of IC, likely mediated by MMP-14. The simultaneous upregulation of circulating sENG and hepatic MMP-14 protein expression in DDC-fed mice supports the involvement of enhanced proteolytic activity in IC, as demonstrated previously [22, 46]. TRC105 increased MMP-14 expression, likely promoting ENG cleavage into sENG, consistent with previous reports that it activates c-Jun N-terminal kinase signalling, upregulates MMP-14, and facilitates ENG–MMP-14 complex formation at the cell surface [47]. This mechanism likely explains the markedly elevated sENG serum concentration observed in our TRC105-treated DDC mice and is in agreement with previous reports of increased sENG following anti-ENG mAb administration [22].

Together, these data suggest that TRC105 is able to restore membrane-bound ENG and enhance its cleavage into sENG via MMP-14. The resulting increase in sENG may antagonize membrane-bound ENG functions, such as promoting monocyte adhesion and transmigration [48], as well as thrombus formation [49].

Furthermore, we examined the consequences of ENG and sENG modulation. The upregulation of inflammatory markers, including p65 NF-κB, ICAM-1, and VCAM-1, observed in DDC-fed mice reflects enhanced endothelial activation and liver inflammation, consistent with the established pathophysiology of IC. These molecules play essential roles in the inflammatory signalling: p65 NF-κB functions as a transcription factor driving the expression of pro-inflammatory genes, while ICAM-1 and VCAM-1 mediate leukocyte adhesion and transmigration, thereby exacerbating local inflammatory responses [50]. Notably, ID1 has been shown to activate the NF-κB pathway through direct interaction with the p65 subunit [51]. This suggests that ID1 may serve as a downstream effector within the BMP-9/ENG/SMAD1/5/9 signalling axis, which is closely associated with inflammation.

The results demonstrate that DDC-fed mice exhibit significantly increased ET-1 protein expression compared with controls, indicating enhanced vasoconstrictive and pro-inflammatory signalling associated with endothelial activation. Elevated ET-1, as a potent vasoconstrictor, can increase intrahepatic vascular resistance and portal pressure, potentially leading to hepatic congestion and fluid retention, which may further exacerbate liver injury and inflammation [52–54]. This is consistent with prior studies linking ET-1 overexpression to endothelial inflammation and dysfunction in liver injury models, including those induced by cholestatic stress [8, 55]. Notably, pharmacological inhibition of the ET-1/endothelin A receptor axis has been shown to decrease hepatic inflammation and fibrosis in mouse models of liver damage [56]. In our study, treatment with the anti-ENG mAb TRC105 significantly prevented ET-1 upregulation, indicating that ENG blockade may reduce endothelial activation and subsequent inflammatory responses in IC, despite not directly targeting ET-1 or its receptors. Together, these findings suggest that TRC105, by stabilizing membrane-bound ENG and modulating endothelial function, may indirectly mitigate ET-1–driven vasoconstriction and the downstream inflammatory cascade, highlighting a mechanistic link between ENG signalling, endothelial homeostasis, and ET-1–mediated vascular and inflammatory responses in cholestatic liver injury. Indeed, treatment with TRC105 significantly prevented the upregulation of p65 NF-κB, ICAM-1, VCAM-1, as well as ET-1 and the downregulation of p-eNOS in DDC mice. In addition, TRC105 prevented upregulation of biomarkers of liver inflammation and macrophage activation, including *Ccl2*, *Ly6c*, *Il-1β*, and *Tnf-α* [35]. This suggests that ENG blockade may help preserve endothelial function and suppress hepatic inflammation. Supporting this interpretation, studies in cholestatic rats have shown that elevated NF-κB activity suppresses eNOS, while NF-κB inhibition restores eNOS function and reduces hepatic inflammation [57].

In murine cholestasis models, Galectin-3 in hepatic macrophages promotes proinflammatory signalling and inflammasome activation, whereas its deficiency mitigates inflammation and disease progression [58, 59]. CD11b -positive macrophages are similarly linked to activation and recruitment of proinflammatory myeloid cells (monocytes) in liver injury, reflecting changes in macrophage functional phenotype during inflammation [60, 61]. In this study, TRC105 reduced activation-associated macrophage markers (Galectin-3 and infiltration CD11b) in cholestatic mice, suggesting the anti-inflammatory potential of anti-ENG mAb treatment.

The question is whether modulation of ENG/SMADs signalling is related to these potentially beneficial effects during IC after TRC105 treatment. DDC feeding significantly increased phosphorylated SMAD1/5/9 and its downstream target ID1, as well as phosphorylated SMAD2/3 and PAI-1. This shows activation of both BMP (SMAD1/5/9–ID1) and TGF-β (SMAD2/3–PAI-1) signalling pathways mediated by ENG [62]. The SMAD1/5/9–ID1 axis, downstream of BMP signalling via ENG, modulates inflammatory responses and promotes tissue repair to maintain vascular integrity [14, 15]. However, its dysregulation may primarily initiate inflammation, which can subsequently contribute to fibrotic remodelling in chronic liver disease [16, 17]. In contrast, the SMAD2/3 PAI-1 pathway, driven by TGF-β, primarily induces fibrotic responses that, in turn, exacerbate inflammation by enhancing matrix deposition and recruiting inflammatory cells [18, 19]. Reduced ENG expression in DDC-fed mice might disrupt the balance within the TGF-β superfamily signalling network by favouring increased activation of the SMAD2/3 pathway. We observed a significant 26% increase in the p-SMAD2/3 to p-SMAD1/5/9 ratio in DDC mice compared to controls, strongly indicating a shift toward SMAD2/3-dominant signalling (Fig. 6A and B). This axis is well established as a key driver of fibrogenesis and inflammation in cholestatic liver diseases [63]. Consistent with our findings, an independent study reported rapid and simultaneous activation of NF-κB and SMAD2/3 pathways in cholestatic hepatocytes, supporting the involvement of SMAD2/3 in the inflammatory response during cholestasis [64], highlighting the broader pathological implications of SMAD2/3 upregulation in IC.

In our previous study, we also established that the mouse anti-ENG mAb M1043 prevented the upregulation of p-SMAD1/5/ID1, suggesting potential anti-inflammatory effects in a mouse model of MASH [65]. Consistent with our results, Tripska et al.. study demonstrated that TRC105 pretreatment decreased phosphorylation of both SMAD1/5/9 and SMAD2/3 in response to 7-ketocholesterol- and high glucose-induced endothelial dysfunction in human aortic endothelial cells [66]. These findings suggest that TRC105’s ability to prevent activation of both signalling pathways, potentially by modulating ENG’s function as a co-receptor in TGF-β and BMP9 signalling [67].

**Fig. 8:**
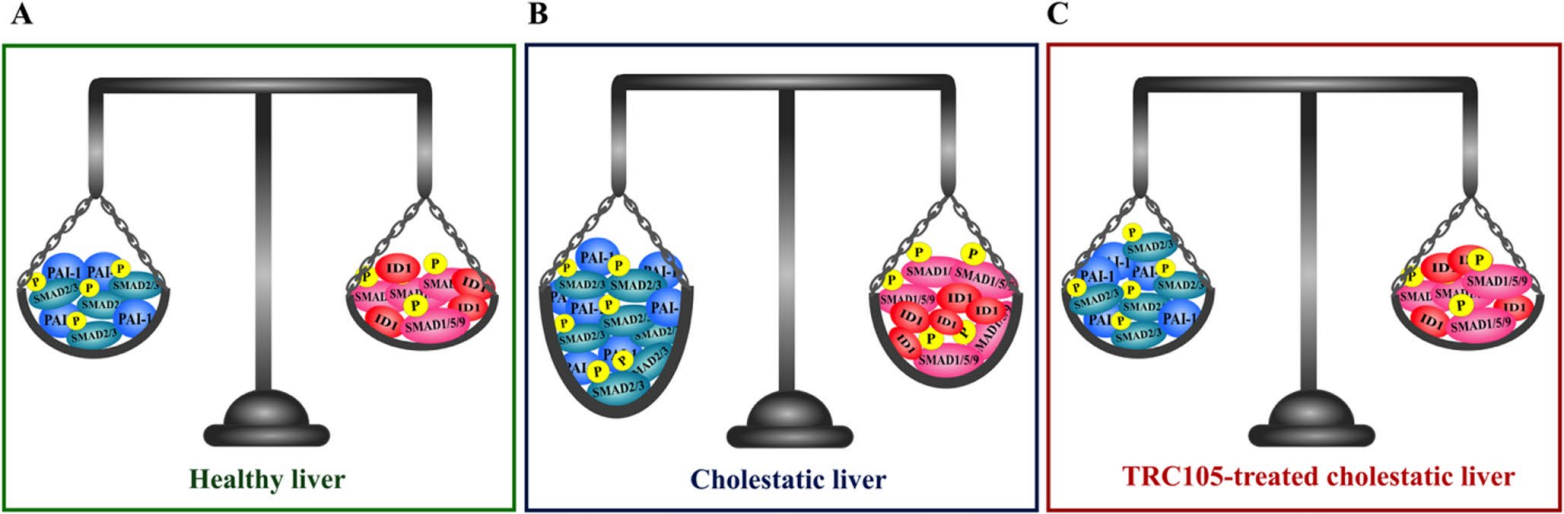
Modulation of SMADs signalling by TRC105 in cholestatic liver injury. Physiological condition: ENG expressed at normal levels, ensuring balanced BMP (SMAD1/5/9/ID1) and TGF-β (SMAD2/3/PAI-1) signalling (**A**). Cholestatic liver (IC model): reduced ENG expression disrupts signalling balance, favouring SMAD2/3 overactivation, promoting inflammation and fibrosis (**B**). TRC105 treatment: restore ENG expression, rebalance SMADs signalling, and mitigate pathological SMAD2/3 dominance (**C**)

In this study, treatment with TRC105 resulted in an approximately 24% reduction in the p-SMAD2/3 to p-SMAD1/5/9 ratio in IC mice, indicating a restoration of balance between these signalling pathways and a potential decrease in disease progression (Fig. 8). This effect may highlight TRC105’s ability to restore ENG function and modulate SMADs signalling, suggesting a dual role in preserving endothelial function and reducing inflammation in cholestatic liver injury.

Taken together, our results demonstrate that TRC105 treatment prevents the upregulation of inflammatory markers, macrophage infiltration, and activation, suggesting its potential to mitigate endothelial activation and the associated inflammatory cascade. These findings align with the conclusion of our previous paper, emphasizing the critical role of anti-ENG mAb treatment in modulating ENG signalling in LSECs with respect to inflammation prevention during MASH [22]. 

### Conclusion

This study demonstrates for the first time that TRC105, an anti-ENG mAb, prevents the increase of inflammatory biomarkers reflecting LSECs activation and the hepatic inflammation in a DDC-induced mouse model of IC, by modulating ENG expression and rebalancing the SMADs signalling pathways. These findings lay the groundwork for considering ENG as a promising pharmacological target for the treatment of inflammation in cholestatic liver diseases.

### Limitations and future directions

The study’s 4-week treatment duration may have been insufficient to affect fibrosis progression or biochemical markers, highlighting the need for longer-term studies. Future research should explore the efficacy of TRC105 in other cholestatic models and assess dose–response relationships. Additionally, conducting in vitro studies using primary liver cell cultures could clarify TRC105’s direct effects on endothelial signalling and inflammation, complementing the in vivo findings. Evaluating combination therapies that target fibrogenesis, such as Farnesoid X receptor agonists (e.g., obeticholic acid) [68], could further enhance TRC105’s therapeutic potential in chronic liver diseases.

## Supplementary information

Supplementary material.

Supplementary Material 1

Supplementary Material 2

Supplementary Material 3

Supplementary Material 4

## Data Availability

The original contributions of this study are detailed in the article; further inquiries can be directed to the corresponding author.

## Abbreviations

Acta2: Actin alpha 2
ALT: Alanine transaminase
ALP: Alkaline phosphatase
Ccl2: C-C motif chemokine ligand 2
Col1a1: Collagen type I alpha 1 chain
DDC: 3,5-diethoxycarbonyl-1,4-dihydrocollidine
ECM: Extracellular matrix
ENG: Endoglin
ET-1: Endothelin-1
Gapdh: Glyceraldehyde 3-phosphate dehydrogenase
HSCs: Hepatic stellate cells
IC: Intrahepatic cholestasis
ICAM-1: Intercellular adhesion molecule-1
ID1: Inhibitor of DNA binding 1
IHC: Immunohistochemical
LSECs: Liver sinusoidal endothelial cells
LSED: Liver sinusoidal endothelial dysfunction
Ly6c: Lymphocyte antigen 6 complex, locus C
MASH: Metabolic dysfunction-associated steatohepatitis
mAb: Monoclonal antibody
MMP-14: Matrix metalloproteinase-14
NF-κB: Nuclear factor kappa B
NLRP3: NLR family pyrin domain-containing 3
PAI-1: Plasminogen activator inhibitor-1
p-eNOS: Phosphorylated endothelial nitric oxide synthase
Pdgfb: Platelet-derived growth factor subunit B
qRT-PCR: Quantitative reverse transcription-polymerase chain reaction
sENG: Soluble endoglin
TBS-T: Tris-buffered saline containing 0.1% Tween-20
TGF-β: Transforming growth factor β
TNF-α: Tumour necrosis factor-alpha
VCAM-1: Vascular cell adhesion molecule-1

## References

[1] Trauner M, Meier PJ, Boyer JL (1998) Molecular pathogenesis of cholestasis. N Engl J Med 339:1217–1227 9780343 10.1056/NEJM199810223391707

[2] Sherlock S, Dooley JS (2008) Diseases of the liver and biliary system. Wiley

[3] Trauner M, Boyer JL (2003) Bile salt transporters: molecular characterization, function, and regulation. Physiol Rev 83:633–671 12663868 10.1152/physrev.00027.2002

[4] dela Peña A, Leclercq I, Field J, George J, Jones B, Farrell G (2005) NF-κB activation, rather than TNF, mediates hepatic inflammation in a murine dietary model of steatohepatitis. Gastroenterology 129:1663–1674 16285964 10.1053/j.gastro.2005.09.004

[5] Zhong L, Simard MJ, Huot J (2018) Endothelial microRNAs regulating the NF-κB pathway and cell adhesion molecules during inflammation. FASEB J 32:4070–4084 29565737 10.1096/fj.201701536R

[6] Arsenijevic A, Stojanovic B, Milovanovic J, Arsenijevic D, Arsenijevic N, Milovanovic M (2020) Galectin-3 in inflammasome activation and primary biliary cholangitis development. Int J Mol Sci 21:5097 32707678 10.3390/ijms21145097 PMC7404314

[7] Vicen M, Igreja Sa IC, Tripska K, Vitverova B, Najmanova I, Eissazadeh S, Micuda S, Nachtigal P (2020) Membrane and soluble endoglin role in cardiovascular and metabolic disorders related to metabolic syndrome. Cell Mol Life Sci 78(6):2405–2418 33185696 10.1007/s00018-020-03701-w PMC11072708

[8] Eissazadeh S, Mohammadi S, Faradonbeh FA, Rathouska JU, Nemeckova I, Tripska K, Vitverova B, Dohnalkova E, Vasinova M, Fikrova P, Sa ICI, Micuda S, Nachtigal P (2024) Endoglin and soluble endoglin in liver sinusoidal endothelial dysfunction in vivo. Biochim Biophys Acta Mol Basis Dis 1870:166990 38110128 10.1016/j.bbadis.2023.166990

[9] St-Jacques S, Cymerman U, Pece N, Letarte M (1994) Molecular characterization and in situ localization of murine endoglin reveal that it is a transforming growth factor-beta binding protein of endothelial and stromal cells. Endocrinology 134:2645–2657 8194490 10.1210/endo.134.6.8194490

[10] Meurer S, Wimmer AE, Leur EV, Weiskirchen R (2019) Endoglin Trafficking/Exosomal Targeting in Liver Cells Depends on N-Glycosylation. Cells 8(9):997 10.3390/cells8090997 PMC6769735 31466384

[11] Lastres P, Bellon T, Cabanas C, Sanchez-Madrid F, Acevedo A, Gougos A, Letarte M, Bernabeu C (1992) Regulated expression on human macrophages of endoglin, an Arg-Gly-Asp-containing surface antigen. Eur J Immunol 22:393–397 1537377 10.1002/eji.1830220216

[12] Meurer SK, Alsamman M, Scholten D, Weiskirchen R (2014) Endoglin in liver fibrogenesis: Bridging basic science and clinical practice. World J Biol Chem 5:180–203 24921008 10.4331/wjbc.v5.i2.180 PMC4050112

[13] Lebrin F, Goumans MJ, Jonker L, Carvalho RL, Valdimarsdottir G, Thorikay M, Mummery C, Arthur HM, and, Dijke P (2004) Endoglin promotes endothelial cell proliferation and TGF-beta/ALK1 signal transduction. EMBO J 23:4018–4028 15385967 10.1038/sj.emboj.7600386 PMC524335

[14] Gadomski S, Singh SK, Singh S, Sarkar T, Klarmann KD, Berenschot M, Seaman S, Jakubison B, Gudmundsson KO, Lockett S (2020) Id1 and Id3 maintain steady-state hematopoiesis by promoting sinusoidal endothelial cell survival and regeneration. Cell reports 31 10.1016/j.celrep.2020.107572 PMC8459380 32348770

[15] Zhang N, Subbaramaiah K, Yantiss RK, Zhou XK, Chin Y, Scherl EJ, Bosworth BP, Benezra R, Dannenberg AJ (2015) Id1 expression in endothelial cells of the colon is required for normal response to injury. Am J Pathol 185:2983–2993 26348574 10.1016/j.ajpath.2015.07.005 PMC4630175

[16] Herrera B, Addante A, Sánchez A (2017) BMP Signalling at the crossroad of liver fibrosis and regeneration. Int J Mol Sci 19(1):39 10.3390/ijms19010039 PMC5795989 29295498

[17] Wiercinska E, Wickert L, Denecke B, Said HM, Hamzavi J, Gressner A, Thorikay M, ten Dijke P, Mertens PR, Breitkopf K (2006) Id1 is a critical mediator in TGF-β–induced transdifferentiation of rat hepatic stellate cells. Hepatology 43:1032–1041 16628634 10.1002/hep.21135

[18] Fabregat I, Moreno-Càceres J, Sánchez A, Dooley S, Dewidar B, Giannelli G, Dijke T, P (2016) TGF-β signalling and liver disease. Febs j 283:2219–2232 26807763 10.1111/febs.13665

[19] Dooley S, ten Dijke P (2012) TGF-β in progression of liver disease. Cell Tissue Res 347:245–256 22006249 10.1007/s00441-011-1246-y PMC3250614

[20] Hu J, Liu Y, Pan Z, Huang X, Wang J, Cao W, Chen Z (2023) Eupatilin ameliorates hepatic fibrosis and hepatic stellate cell activation by suppressing β-catenin/PAI-1 Pathway. Int J Mol Sci 24(6):5933 10.3390/ijms24065933 PMC10054508 36983006

[21] Aso Y (2007) Plasminogen activator inhibitor (PAI)-1 in vascular inflammation and thrombosis. Front Biosci 12:2957–2966 17485272 10.2741/2285

[22] Eissazadeh S, Fikrova P, Rathouska JU, Nemeckova I, Tripska K, Vasinova M, Havelek R, Mohammadi S, Sa I, Theuer IC, Konig C, Micuda M, S., and, Nachtigal P (2025) Anti-Endoglin monoclonal antibody prevents the progression of liver sinusoidal endothelial inflammation and fibrosis in MASH. Life Sci 364:123428 39889923 10.1016/j.lfs.2025.123428

[23] Sa CI, Tripska I, Alaei Faradonbeh K, Hroch F, Lastuvkova M, Schreiberova H, Kacerovsky J, Pericacho M, Nachtigal M, P., and, Micuda S (2023) Labetalol and soluble endoglin aggravate bile acid retention in mice with ethinylestradiol-induced cholestasis. Front Pharmacol 14:1116422 36778021 10.3389/fphar.2023.1116422 PMC9909014

[24] Park ES, Kim S, Yao DC, Savarraj JPJ, Choi HA, Chen PR, Kim E (2022) Soluble endoglin stimulates inflammatory and angiogenic responses in microglia that are associated with endothelial dysfunction. Int J Mol Sci 23(3):1225 10.3390/ijms23031225 PMC8835690 35163148

[25] Varejckova M, Gallardo-Vara E, Vicen M, Vitverova B, Fikrova P, Dolezelova E, Rathouska J, Prasnicka A, Blazickova K, Micuda S, Bernabeu C, Nemeckova I, Nachtigal P (2017) Soluble endoglin modulates the pro-inflammatory mediators NF-kappaB and IL-6 in cultured human endothelial cells. Life Sci 175:52–60 28336397 10.1016/j.lfs.2017.03.014

[26] Nolan-Stevaux O, Zhong W, Culp S, Shaffer K, Hoover J, Wickramasinghe D, Ruefli-Brasse A (2012) Endoglin requirement for BMP9 signaling in endothelial cells reveals new mechanism of action for selective anti-endoglin antibodies. PLoS ONE 7:e50920 23300529 10.1371/journal.pone.0050920 PMC3531442

[27] Liu Y, Tian H, Blobe GC, Theuer CP, Hurwitz HI, Nixon AB (2014) Effects of the combination of TRC105 and bevacizumab on endothelial cell biology. Invest New Drugs 32:851–859 24994097 10.1007/s10637-014-0129-y PMC4169868

[28] Liu Y, Starr MD, Brady JC, Dellinger A, Pang H, Adams B, Theuer CP, Lee NY, Hurwitz HI, Nixon AB (2014) Modulation of circulating protein biomarkers following TRC105 (anti-endoglin antibody) treatment in patients with advanced cancer. Cancer Med 3:580–591 24574330 10.1002/cam4.207 PMC4101749

[29] Rosen LS, Hurwitz HI, Wong MK, Goldman J, Mendelson DS, Figg WD, Spencer S, Adams BJ, Alvarez D, Seon BK, Theuer CP, Leigh BR, Gordon MS (2012) A phase I first-in-human study of TRC105 (Anti-Endoglin Antibody) in patients with advanced cancer. Clin Cancer Res 18:4820–4829 22767667 10.1158/1078-0432.CCR-12-0098 PMC3432706

[30] Kumar S, Pan CC, Bloodworth JC, Nixon AB, Theuer C, Hoyt DG, Lee NY (2014) Antibody-directed coupling of endoglin and MMP-14 is a key mechanism for endoglin shedding and deregulation of TGF-beta signaling. Oncogene 33:3970–3979 24077288 10.1038/onc.2013.386 PMC3969897

[31] Pose E, Sancho-Bru P, Coll M (2019) 3,5-Diethoxycarbonyl-1,4-Dihydrocollidine Diet: A Rodent Model in Cholestasis Research. Methods Mol Biol 1981, 249–257 10.1007/978-1-4939-9420-5_16 31016659

[32] Graham ML, Prescott MJ (2015) The multifactorial role of the 3Rs in shifting the harm-benefit analysis in animal models of disease. Eur J Pharmacol 759:19–29 25823812 10.1016/j.ejphar.2015.03.040 PMC4441106

[33] Kolouchova G, Brcakova E, Hirsova P, Sispera L, Tomsik P, Cermanova J, Hyspler R, Slanarova M, Fuksa L, Lotkova H (2011) Pravastatin modulates liver bile acid and cholesterol homeostasis in rats with chronic cholestasis. J Gastroenterol Hepatol 26:1544–1551 21501227 10.1111/j.1440-1746.2011.06748.x

[34] Eissazadeh S, Rathouska JU, Nemeckova I, Fikrova P, Tripska K, Vasinova M, Kolackova M, Nachtigal P (2025) Cost-effective method for semi-quantitative analysis of soluble endoglin in biological samples after anti-endoglin monoclonal antibody treatment. Sci Rep 15:38066 41168366 10.1038/s41598-025-21972-w PMC12575752

[35] Lastuvkova H, Dohnalkova E, Manna DF, Cermanova J, Mokry J, Pejchal J, Hirsova P, Nachtigal P, Pavkova I, Bajnokova M, Smutna L, Stefela A, Kamaraj R, Jandova L, Uher M, Pavek P, Micuda S, Hroch M (2025) Dimethyl fumarate attenuates bile acid retention and liver fibrosis in a mouse model of cholestasis. Am J Physiol gastrointest liver physiol 10.1152/ajpgi.00262.2024 40210415

[36] Gijbels E, Pieters A, De Muynck K, Vinken M, Devisscher L (2021) Rodent models of cholestatic liver disease: A practical guide for translational research. Liver Int 41:656–682 33486884 10.1111/liv.14800 PMC8048655

[37] Fickert P, Stoger U, Fuchsbichler A, Moustafa T, Marschall HU, Weiglein AH, Tsybrovskyy O, Jaeschke H, Zatloukal K, Denk H, Trauner M (2007) A new xenobiotic-induced mouse model of sclerosing cholangitis and biliary fibrosis. Am J Pathol 171:525–536 17600122 10.2353/ajpath.2007.061133 PMC1934539

[38] Finnson KW, Philip A (2012) Endoglin in liver fibrosis. J Cell Commun Signal 6:1–4 22131199 10.1007/s12079-011-0154-y PMC3271193

[39] Sun J, Wang J, Zhang N, Yang R, Chen K, Kong D (2019) Identification of global mRNA expression profiles and comprehensive bioinformatic analyses of abnormally expressed genes in cholestatic liver disease. Gene 707:9–21 31048068 10.1016/j.gene.2019.04.078

[40] Song C-W, Qiu W, Zhou X-Q, Feng X-C, Chen W-S (2019) Elevated hepatic MDR3/ABCB4 is directly mediated by MiR-378a-5p in human obstructive cholestasis. Eur Rev Med Pharmacol Sci 23(6):2539–2547 10.26355/eurrev_201903_17402 30964181

[41] Li J, Zhu X, Zhang M, Zhang Y, Ye S, Leng Y, Yang T, Kong L, Zhang H (2021) Limb expression 1-like (LIX1L) protein promotes cholestatic liver injury by regulating bile acid metabolism. J Hepatol 75:400–413 33746084 10.1016/j.jhep.2021.02.035

[42] Zhang L, Yang Z, Trottier J, Barbier O, Wang L (2017) Long noncoding RNA MEG3 induces cholestatic liver injury by interaction with PTBP1 to facilitate shp mRNA decay. Hepatology 65:604–615 27770549 10.1002/hep.28882 PMC5258819

[43] Saito T, Bokhove M, Croci R, Zamora-Caballero S, Han L, Letarte M, de Sanctis D, Jovine L (2017) Structural basis of the human endoglin-BMP9 interaction: insights into BMP signaling and HHT1. Cell Rep 19:1917–1928 28564608 10.1016/j.celrep.2017.05.011 PMC5464963

[44] Rossi E, Bernabeu C (2023) Novel vascular roles of human endoglin in pathophysiology. J Thromb Haemost 21:2327–2338 37315795 10.1016/j.jtha.2023.06.007

[45] Schoonderwoerd MJA, Goumans MTH, Hawinkels L (2020) Endoglin: beyond the Endothelium. Biomolecules 10 10.3390/biom10020289 PMC7072477 32059544

[46] Hawinkels LJ, Kuiper P, Wiercinska E, Verspaget HW, Liu Z, Pardali E, Sier CF, and ten, Dijke P (2010) Matrix metalloproteinase-14 (MT1-MMP)-mediated endoglin shedding inhibits tumor angiogenesis. *Cancer Res* 70, 4141–4150 10.1158/0008-5472.CAN-09-4466 20424116

[47] Kumar S, Pan C, Bloodworth J, Nixon A, Theuer C, Hoyt D, Lee N (2014) Antibody-directed coupling of endoglin and MMP-14 is a key mechanism for endoglin shedding and deregulation of TGF-β signaling. Oncogene 33:3970–3979 24077288 10.1038/onc.2013.386 PMC3969897

[48] Rossi E, Sanz-Rodriguez F, Eleno N, Duewell A, Blanco FJ, Langa C, Botella LM, Cabanas C, Lopez-Novoa JM, Bernabeu C (2013) Endothelial endoglin is involved in inflammation: role in leukocyte adhesion and transmigration. Blood J Am Soc Hematol 121:403–415 10.1182/blood-2012-06-435347 23074273

[49] Rossi E, Pericacho M, Kauskot A, Gamella-Pozuelo L, Reboul E, Leuci A, Egido-Turrion C, El Hamaoui D, Marchelli A, Fernández FJ (2023) Soluble endoglin reduces thrombus formation and platelet aggregation via interaction with αIIbβ3 integrin. J Thromb Haemost 21:1943–1956 36990159 10.1016/j.jtha.2023.03.023

[50] Sucajtys-Szulc E, Debska-Slizien A, Rutkowski B, Szolkiewicz M, Swierczynski J, Smolenski RT (2022) Hepatocyte nuclear factor 1alpha proinflammatory effect linked to the overexpression of liver nuclear factor-kappab in experimental model of chronic kidney disease. Int J Mol Sci 23(16):8883 10.3390/ijms23168883 PMC9408856 36012158

[51] Peng X, Wang Y, Kolli S, Deng J, Li L, Wang Z, Raj JU, Gou D (2012) Physical and functional interaction between the ID1 and p65 for activation of NF-κB. Am J Physiology-Cell Physiol 303:C267–C277 10.1152/ajpcell.00365.2011 PMC3423031 22592405

[52] Chan CC, Wang SS, Lee FY, Chang FY, Lin HC, Hou MC, Huang HC, Lee SD (2004) Effects of endothelin-1 on portal-systemic collaterals of common bile duct-ligated cirrhotic rats. Eur J Clin Invest 34:290–296 15086361 10.1111/j.1365-2362.2004.01336.x

[53] Kojima H, Sakurai S, Kuriyama S, Yoshiji H, Imazu H, Uemura M, Nakatani Y, Yamao J, Fukui H (2001) Endothelin-1 plays a major role in portal hypertension of biliary cirrhotic rats through endothelin receptor subtype B together with subtype A in vivo. J Hepatol 34:805–811 11451162 10.1016/s0168-8278(01)00045-9

[54] Alam I, Bass NM, Bacchetti P, Gee L, Rockey DC (2000) Hepatic tissue endothelin-1 levels in chronic liver disease correlate with disease severity and ascites. Am J Gastroenterol 95:199–203 10638583 10.1111/j.1572-0241.2000.01684.x

[55] Nagai H, Kato A, Kimura F, Shimizu H, Yoshidome H, Ohtsuka M, Furukawa K, Nozawa S, Yoshitomi H, Mitsuhashi N, Takeuchi D, Suda K, Yoshioka I, Miyazaki M (2010) Endothelin-1 aggravates hepatic ischemia/reperfusion injury during obstructive cholestasis in bile duct ligated mice. J Surg Res 162:46–53 20552721 10.1016/j.jss.2007.07.024

[56] Owen T, Carpino G, Chen L, Kundu D, Wills P, Ekser B, Onori P, Gaudio E, Alpini G, Francis H, Kennedy L (2023) Endothelin Receptor-A Inhibition Decreases Ductular Reaction, Liver Fibrosis, and Angiogenesis in a Model of Cholangitis. Cell Mol Gastroenterol Hepatol 16:513–540 37336290 10.1016/j.jcmgh.2023.06.005 PMC10462792

[57] Balasubramanian V, Mehta G, Jones H, Sharma V, Davies N, Jalan R, Mookerjee R (2017) Post-transcriptional regulation of hepatic DDAH1 with TNF blockade leads to improved eNOS function and reduced portal pressure in cirrhotic rats. Sci Rep 7:17900 29263339 10.1038/s41598-017-18094-3 PMC5738445

[58] Arsenijevic A, Milovanovic J, Stojanovic B, Djordjevic D, Stanojevic I, Jankovic N, Vojvodic D, Arsenijevic N, Lukic ML, Milovanovic M (2019) Gal-3 Deficiency Suppresses Novosphyngobium aromaticivorans Inflammasome Activation and IL-17 Driven Autoimmune Cholangitis in Mice. Front Immunol 10:1309 31231399 10.3389/fimmu.2019.01309 PMC6568238

[59] Tian J, Yang G, Chen HY, Hsu DK, Tomilov A, Olson KA, Dehnad A, Fish SR, Cortopassi G, Zhao B, Liu FT, Gershwin ME, Torok NJ, Jiang JX (2016) Galectin-3 regulates inflammasome activation in cholestatic liver injury. FASEB J 30:4202–4213 27630169 10.1096/fj.201600392RR PMC5102125

[60] Lee CM, Peng HH, Yang P, Liou JT, Liao CC, Day YJ (2017) C-C Chemokine Ligand-5 is critical for facilitating macrophage infiltration in the early phase of liver ischemia/reperfusion injury. Sci Rep 7:3698 28623253 10.1038/s41598-017-03956-7 PMC5473895

[61] Tacke F (2012) Functional role of intrahepatic monocyte subsets for the progression of liver inflammation and liver fibrosis in vivo. Fibrogenesis Tissue Repair 5:S27 23259611 10.1186/1755-1536-5-S1-S27 PMC3368797

[62] Goumans M-J, Liu Z, Ten Dijke P (2009) TGF-β signaling in vascular biology and dysfunction. Cell Res 19:116–127 19114994 10.1038/cr.2008.326

[63] Seyhan H, Hamzavi J, Wiercinska E, Gressner A, Mertens P, Kopp J, Horch R, Breitkopf K, Dooley S (2006) Liver fibrogenesis due to cholestasis is associated with increased Smad7 expression and Smad3 signaling. J Cell Mol Med 10:922–932 17125595 10.1111/j.1582-4934.2006.tb00535.x PMC3933087

[64] Delhove JM, Buckley SM, Perocheau DP, Karda R, Arbuthnot P, Henderson NC, Waddington SN, McKay TR (2017) Longitudinal in vivo bioimaging of hepatocyte transcription factor activity following cholestatic liver injury in mice. Sci Rep 7:41874 28157201 10.1038/srep41874 PMC5291111

[65] Peng X, Wang Y, Kolli S, Deng J, Li L, Wang Z, Raj JU, Gou D (2012) Physical and functional interaction between the ID1 and p65 for activation of NF-kappaB. Am J Physiol Cell Physiol 303:C267–277 22592405 10.1152/ajpcell.00365.2011 PMC3423031

[66] Tripska K, Igreja Sa IC, Vasinova M, Vicen M, Havelek R, Eissazadeh S, Svobodova Z, Vitverova B, Theuer C, Bernabeu C, Nachtigal P (2022) Monoclonal anti-endoglin antibody TRC105 (carotuximab) prevents hypercholesterolemia and hyperglycemia-induced endothelial dysfunction in human aortic endothelial cells. Front Med (Lausanne) 9:845918 36160139 10.3389/fmed.2022.845918 PMC9490272

[67] Schoonderwoerd MJ, Goumans M-JT, Hawinkels LJ (2020) Endoglin: beyond the endothelium. Biomolecules 10:289 32059544 10.3390/biom10020289 PMC7072477

[68] Fiorucci S, Urbani G, Distrutti E, Biagioli M (2025) Obeticholic acid and other farnesoid-X-Receptor (FXR) agonists in the treatment of liver disorders. Pharmaceuticals (Basel) 18(9):1424 10.3390/ph18091424 PMC12472620 41011291

