# Supplementary material for: Anti-endoglin monoclonal antibody TRC105 prevents the increase of liver inflammatory biomarkers in a mouse model of cholestasis
Source: Cell Mol Life Sci. 2026 Apr 29;83(1):255. doi: 10.1007/s00018-026-06212-2 (PMC13272742; doi:10.1007/s00018-026-06212-2)

Figure 1. Original uncropped Western blots of GAPDH (≃36-37 kDa) from the membrane fraction (A) and total liver lysates (B). Full-length blots, including molecular weight markers, are shown.

(A)

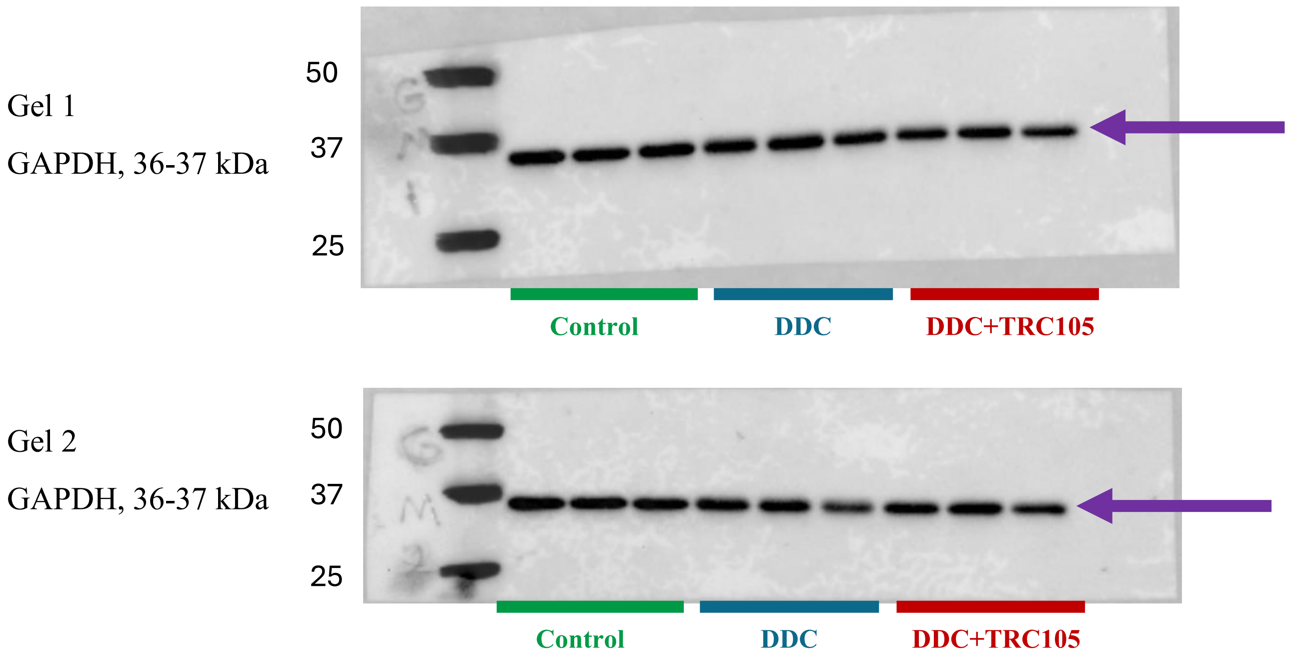

(B)

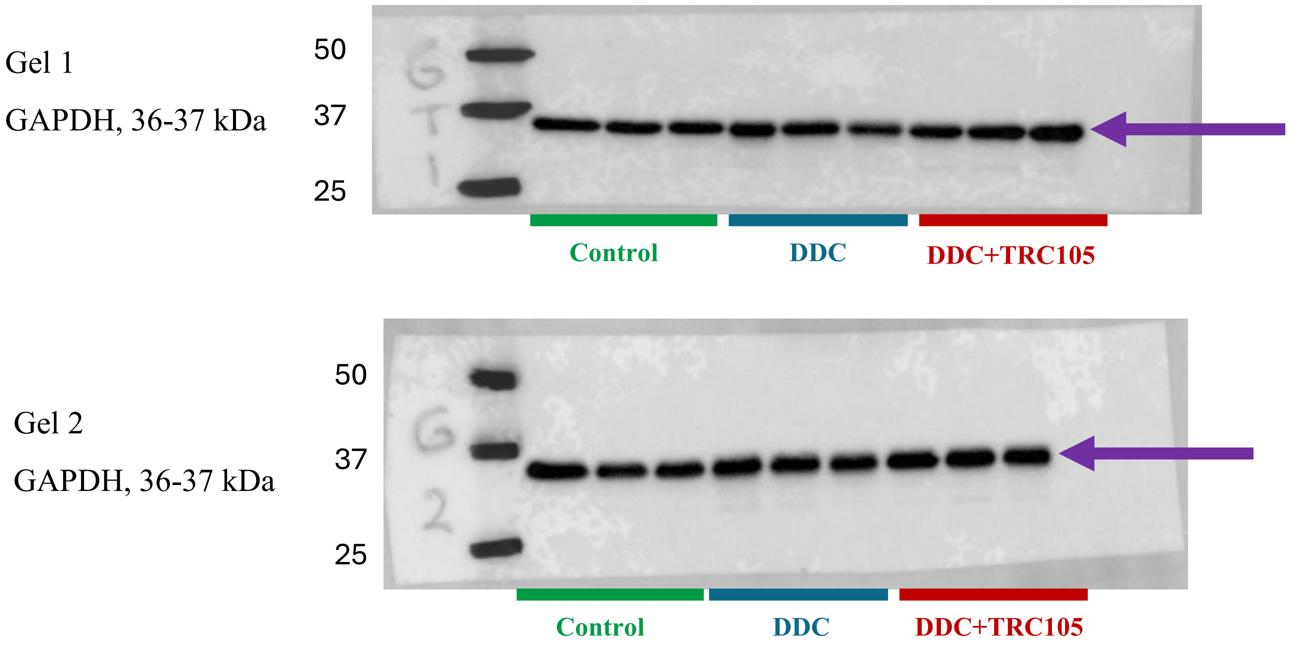

Figure 2. Original uncropped Western blots of ENG (90 kDa). Full-length blots, including molecular weight markers, are shown. Lanes corresponding to the experimental groups are labeled. It represents ENG western blot analysis in Figure 3B.

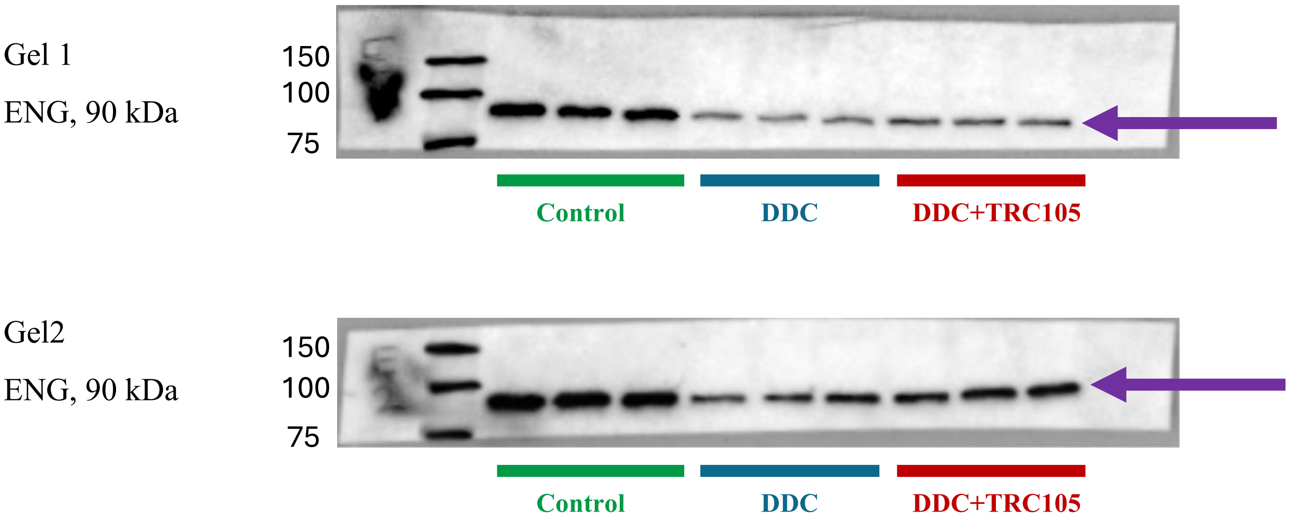

Figure 3. Original uncropped Western blots of p-SMAD1/5/9 (≃60 kDa). Full-length blots, including molecular weight markers, are shown. Lanes corresponding to the experimental groups are labeled. It represents p-SMAD1/5/9 western blot analysis in Figure 3C.

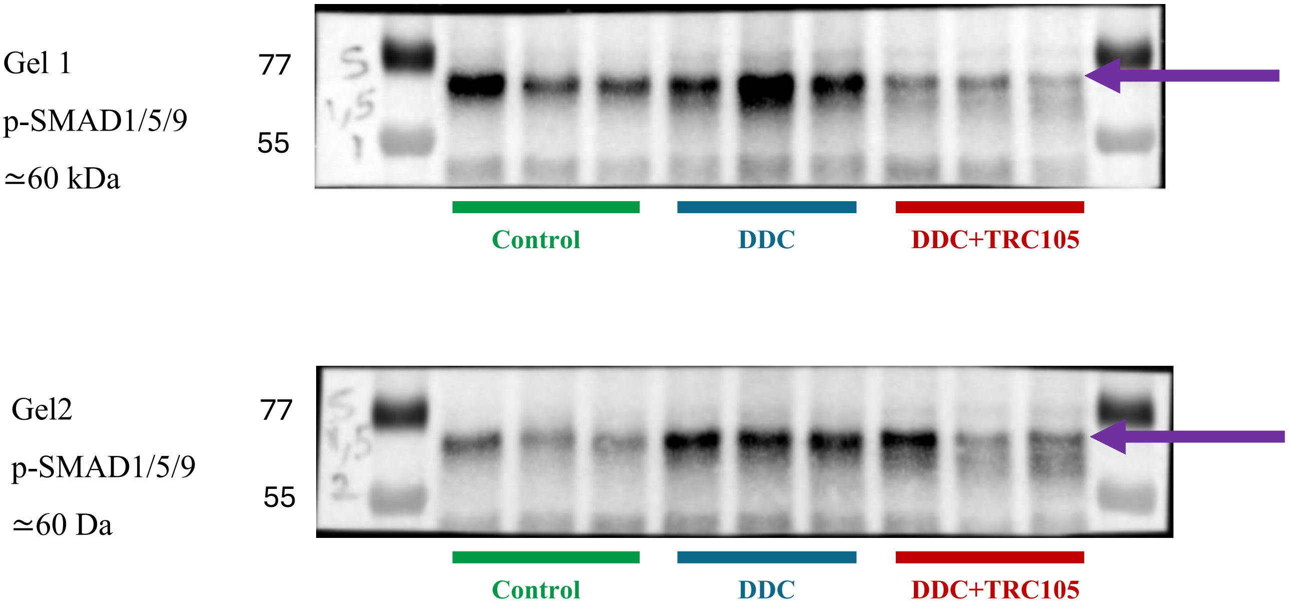

Figure 4. Original uncropped Western blots of ID1 (≃22 kDa). Full-length blots, including molecular weight markers, are shown. Lanes corresponding to the experimental groups are labeled. It represents ID1 western blot analysis in Figure 3D.

Gel 1

ID1, ≃22 kDa

Gel2

ID1, ≃22 kDa

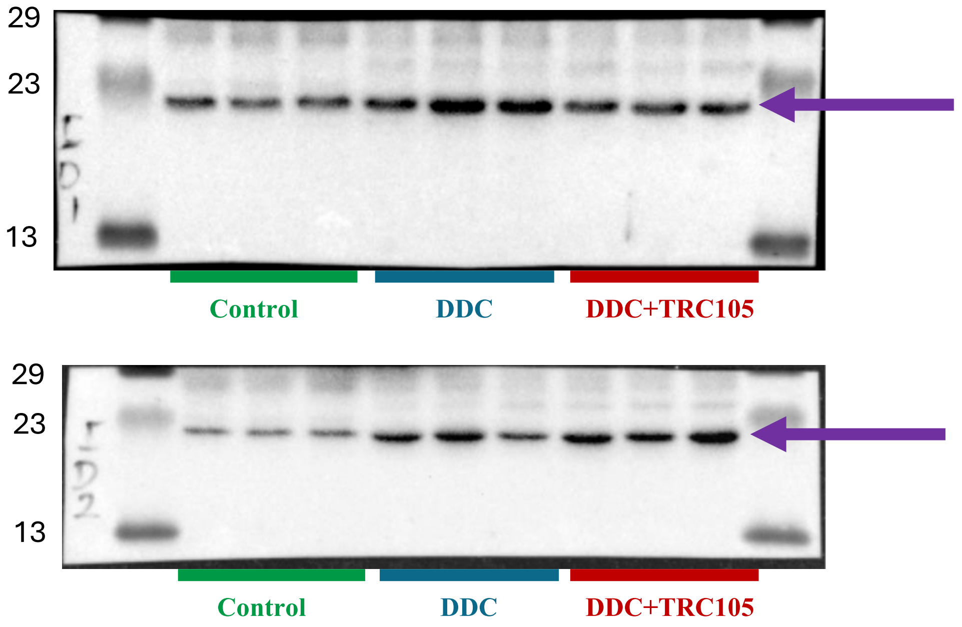

Figure 5. Original uncropped Western blots of p-SMAD2/3 (≃60 kDa). Full-length blots, including molecular weight markers, are shown. Lanes corresponding to the experimental groups are labeled. It represents p-SMAD2/3 western blot analysis in Figure 3E.

Gel 1

p-SMAD2/3

≃60 kDa

Gel 2

p-SMAD2/3

≃60 kDa

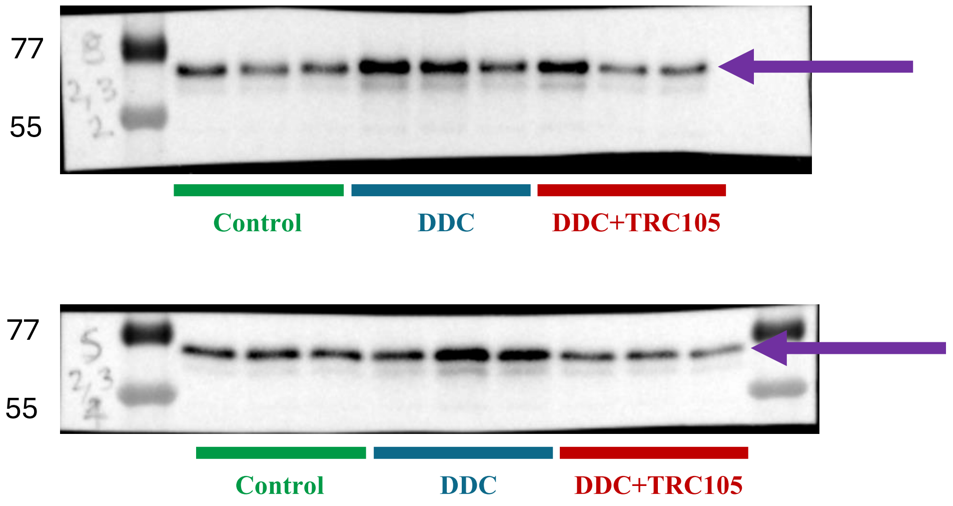

Figure 6. Original uncropped Western blots of PAI-1 (≃40 kDa). Full-length blots, including molecular weight markers, are shown. Lanes corresponding to the experimental groups are labeled. It represents PAI-1 western blot analysis in Figure 3F.

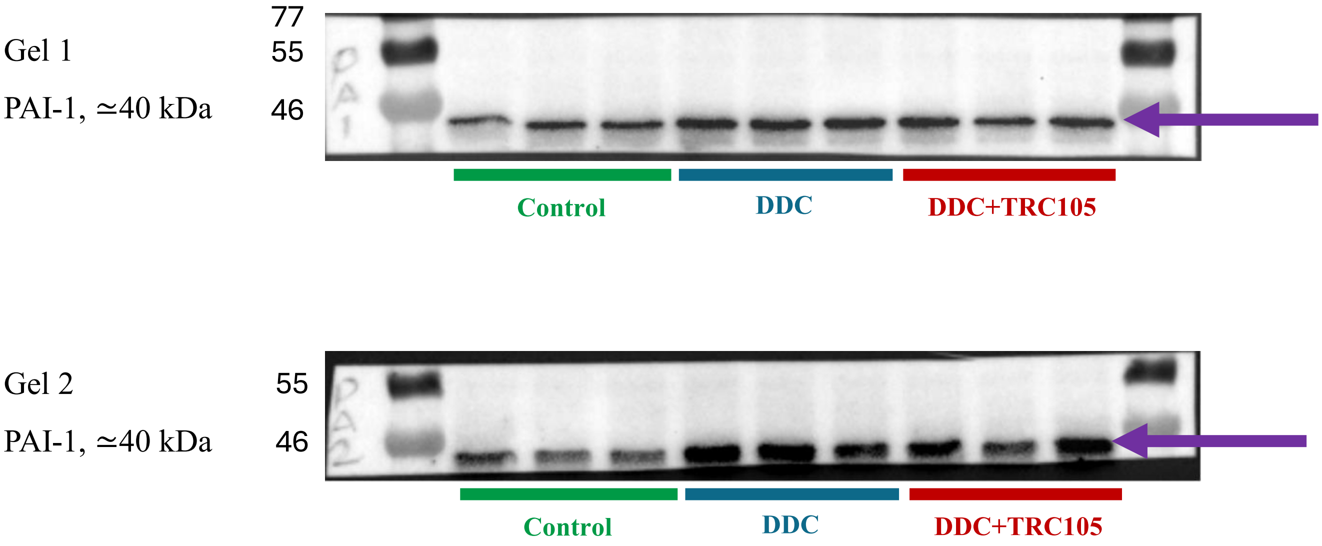

Figure 7. Original uncropped Western blots of p-SMAD2/3 and p/SMAD1/5/9 (≃60 kDa). The full-length blot is shown, including molecular weight markers. It represents p-SMAD2/3 and p/SMAD1/5/9 (control group, DDC group and DDC+TRC105 group) western blot analysis in Figure 3G.

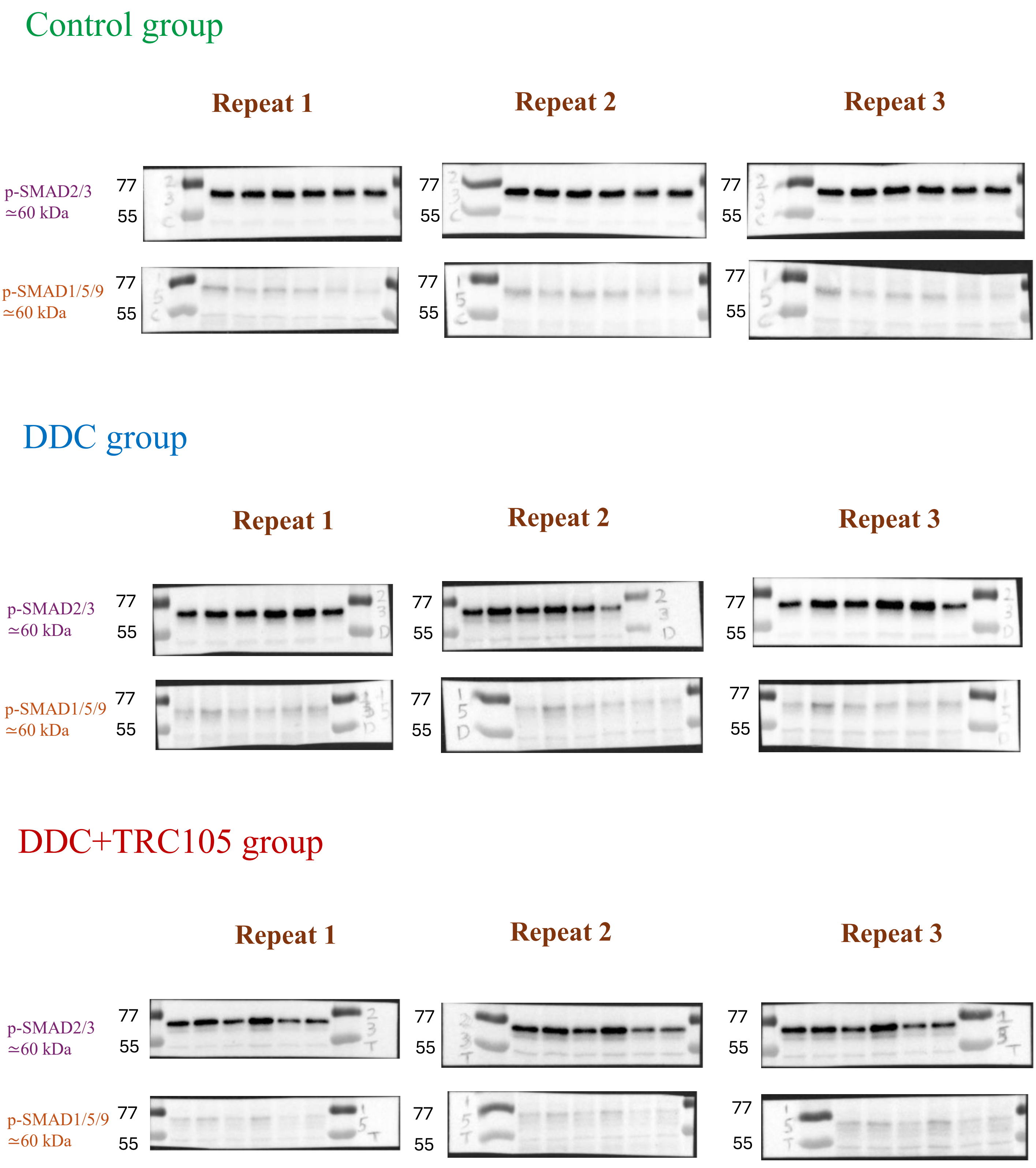

Figure 8. Original uncropped Western blots of MMP-14 (≃60 kDa). Full-length blots, including molecular weight markers, are shown. Lanes corresponding to the experimental groups are labeled. It represents MM-14 western blot analysis in Figure 4A.

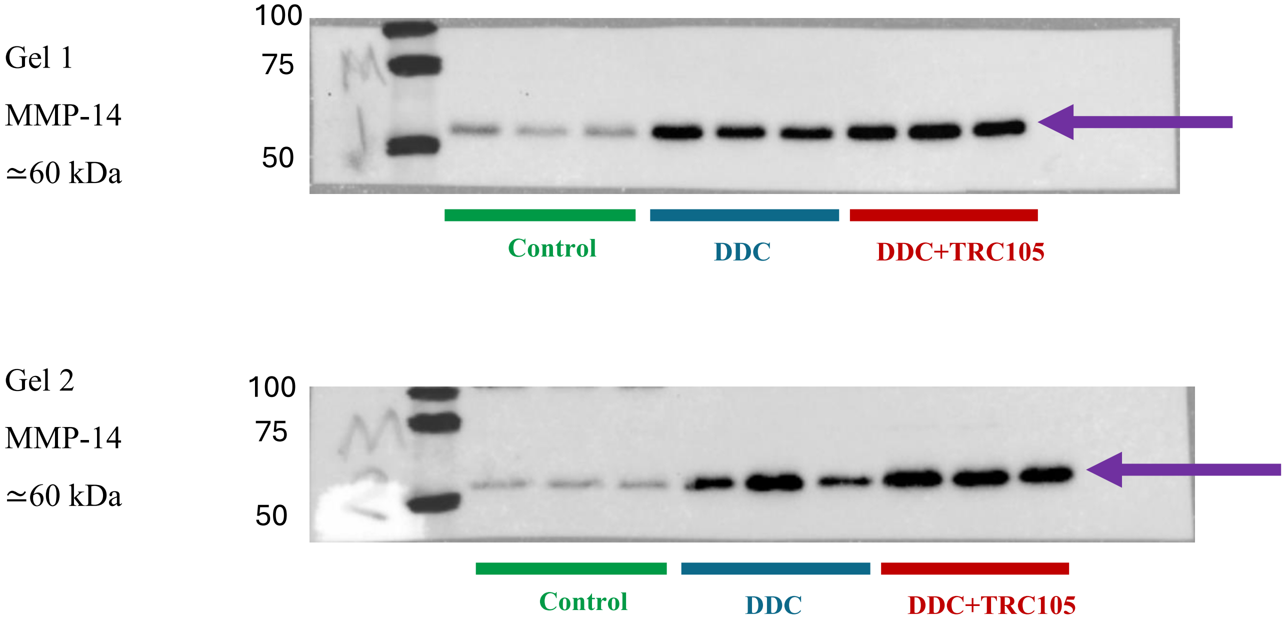

Figure 9. Original uncropped Western blots of sENG (≃77 kDa). Full-length blots, including molecular weight markers, are shown. Lanes corresponding to the experimental groups are labeled. It represents sENG western blot analysis in Figure 4B.

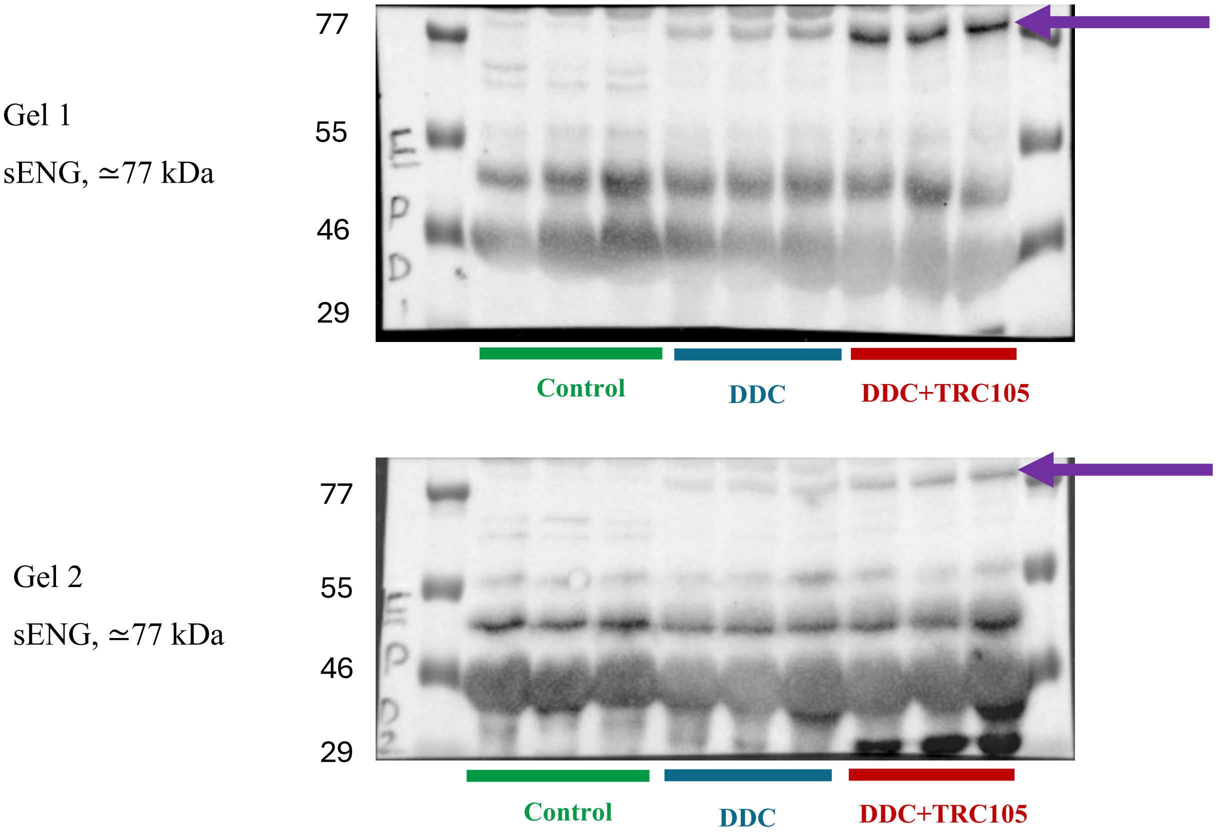

Figure 10. Original uncropped Western blots of p65 NF-κB (≃65 kDa). Full-length blots, including molecular weight markers, are shown. Lanes corresponding to the experimental groups are labeled. It represents p65 NF-κB western blot analysis in Figure 5A.

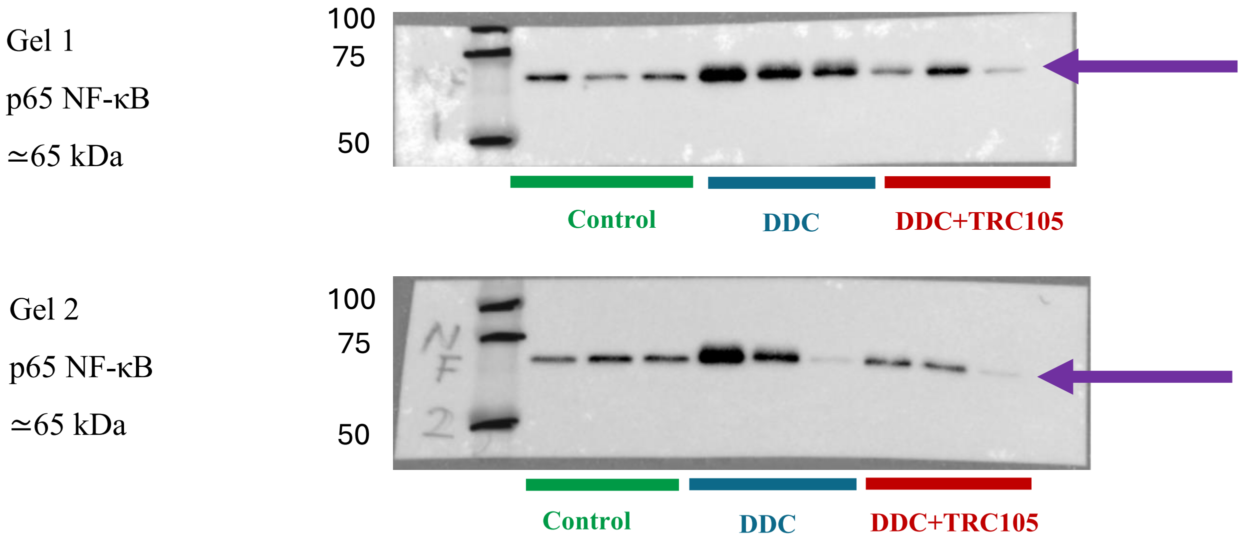

Figure 11. Original uncropped Western blots of ICAM-1 (90 kDa). Full-length blots, including molecular weight markers, are shown. Lanes corresponding to the experimental groups are labeled. It represents ICAM-1 western blot analysis in Figure 5B.

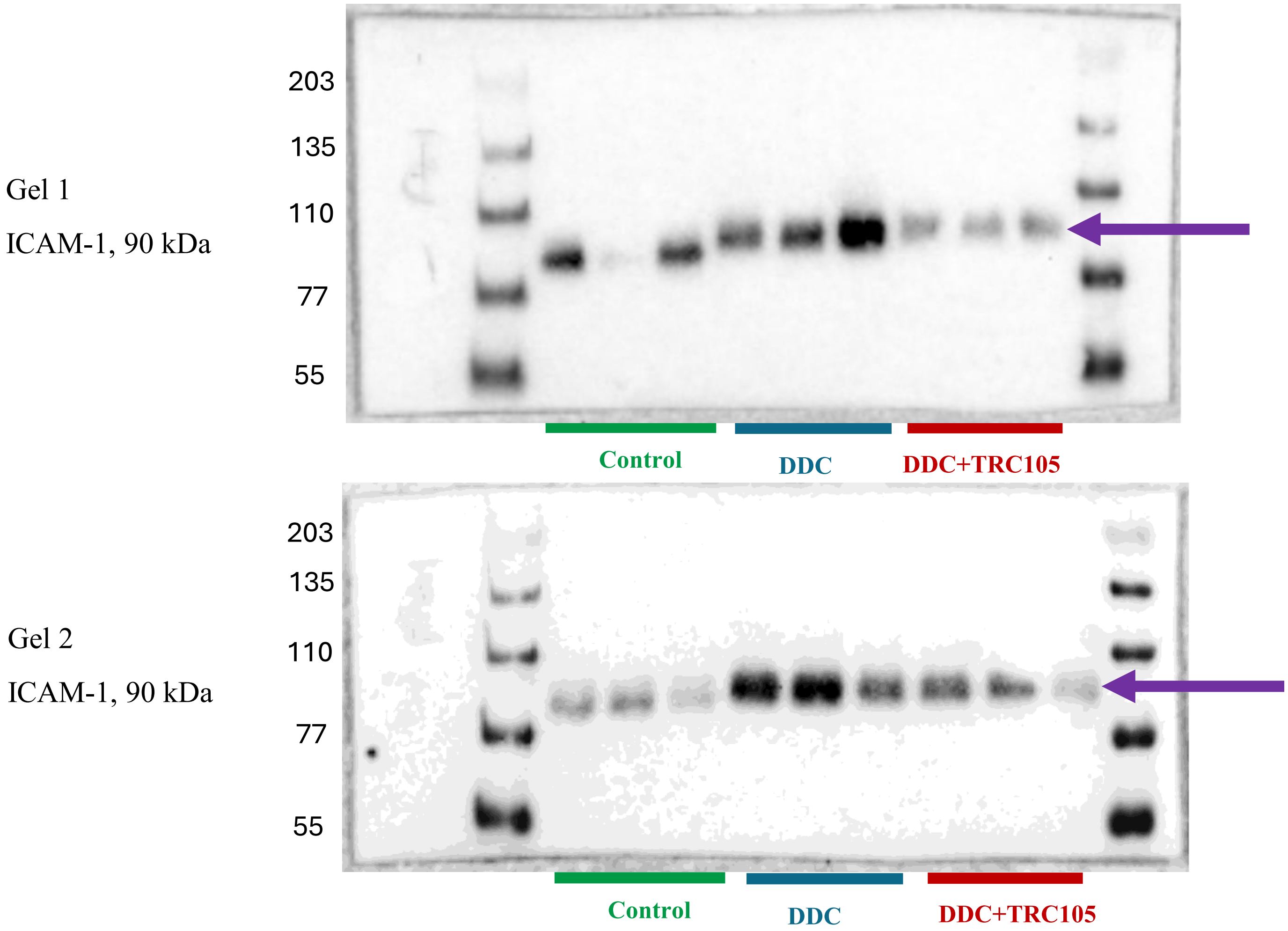

Figure 12. Original uncropped Western blots of VCAM-1 (110 kDa). Full-length blots, including molecular weight markers, are shown. Lanes corresponding to the experimental groups are labeled. It represents VCAM-1 western blot analysis in Figure 5C.

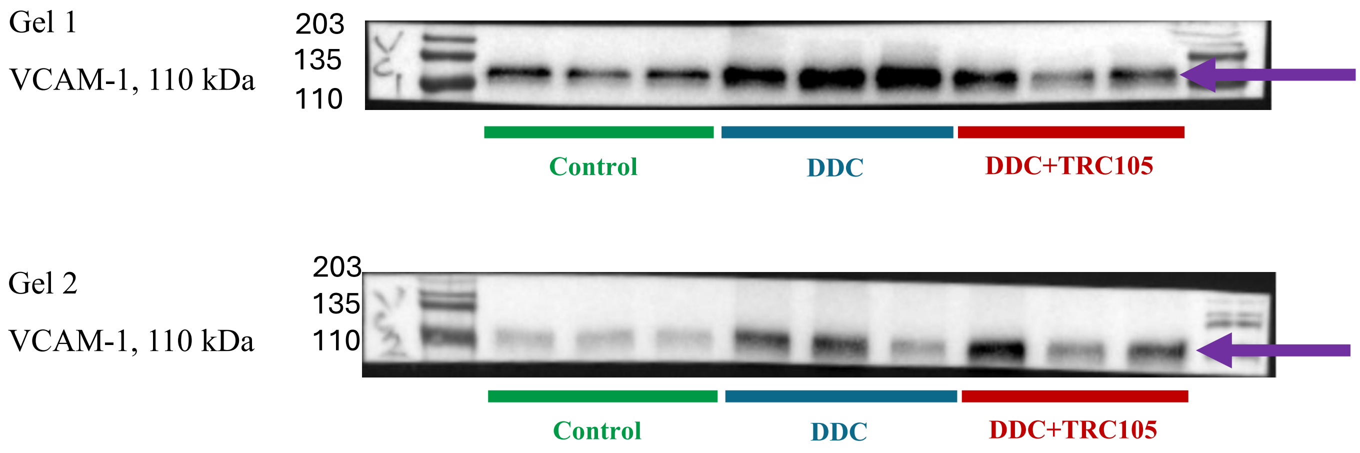

Figure 13. Original uncropped Western blots of ET-1 (≃23 kDa). Full-length blots, including molecular weight markers, are shown. Lanes corresponding to the experimental groups are labeled. It represents ET-1 western blot analysis in Figure 5D.

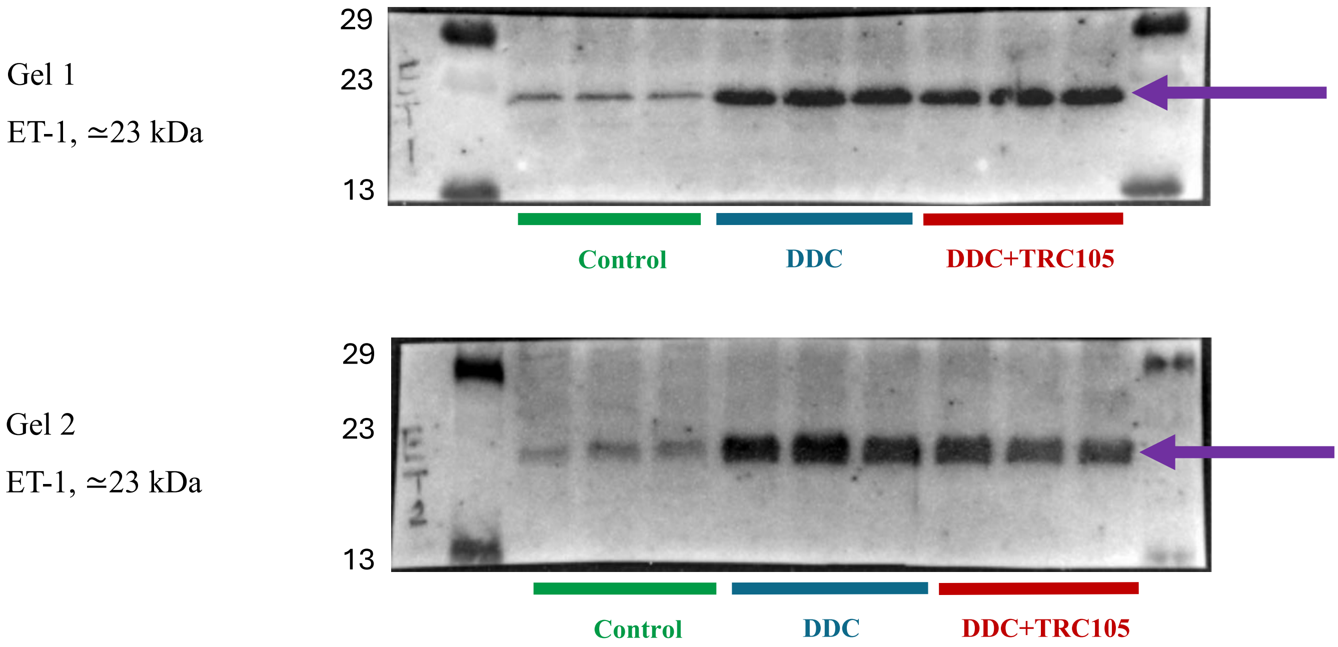

Figure 14. Original uncropped Western blots of p-eNOS (≃130 kDa). Full-length blots, including molecular weight markers, are shown. Lanes corresponding to the experimental groups are labeled. It represents p-eNOS western blot analysis in Figure 5E.

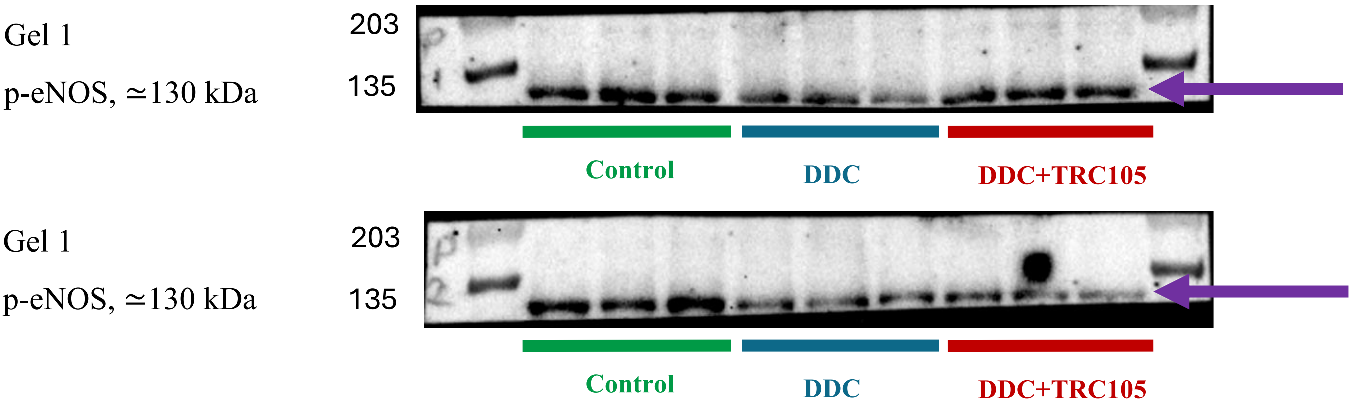

Figure 15. Original uncropped Western blots of eNOS (≃130 kDa). Full-length blots, including molecular weight markers, are shown. Lanes corresponding to the experimental groups are labeled. It represents p-eNOS western blot analysis in Figure 5E.

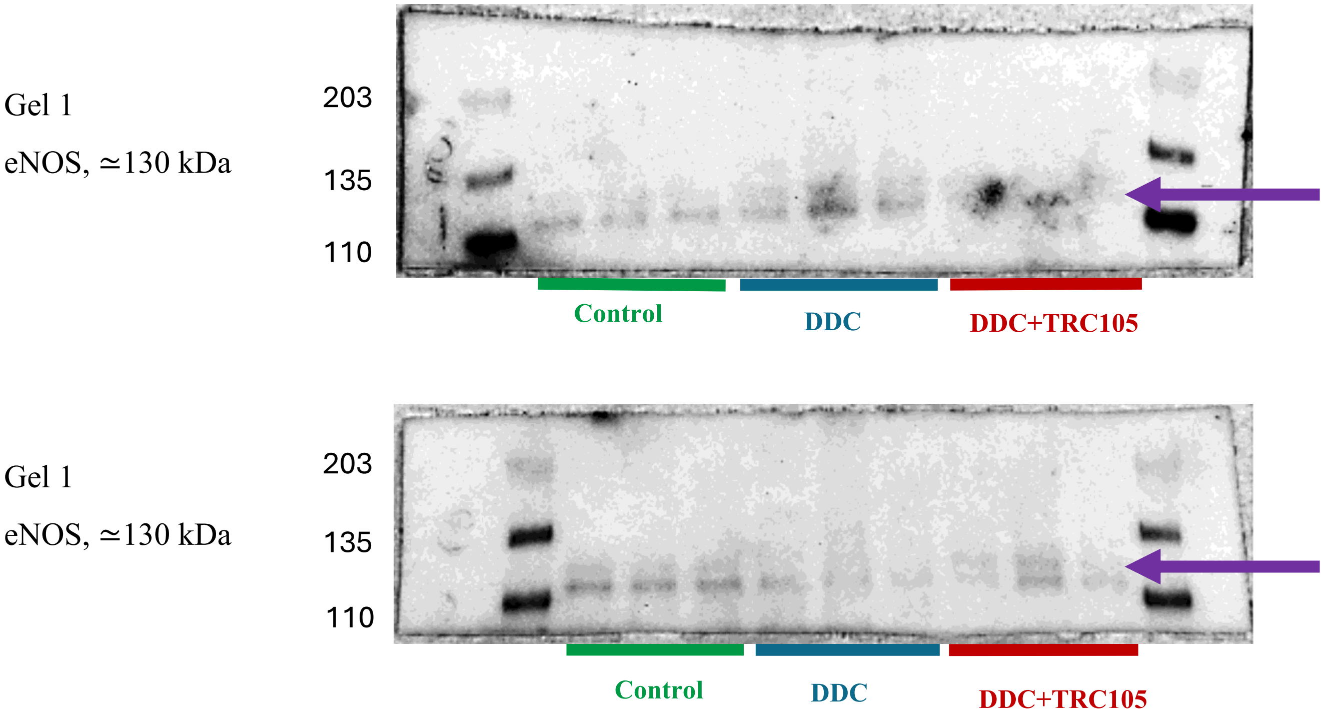

Figure 16. Original uncropped Western blots of Galectin-3 (30 kDa). Full-length blots, including molecular weight markers, are shown. Lanes corresponding to the experimental groups are labeled. It represents Galectin-3 western blot analysis in Figure 6A.

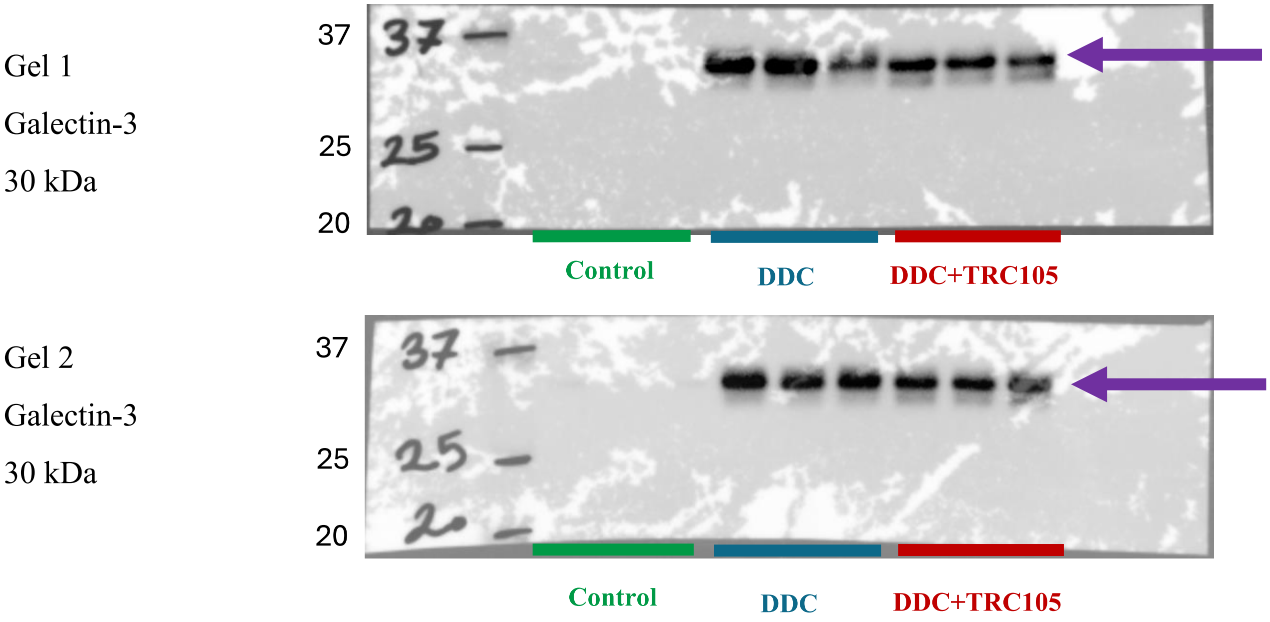

Figure 17. Original uncropped Western blots of CD11b (115-130 kDa). Full-length blots, including molecular weight markers, are shown. Lanes corresponding to the experimental groups are labeled. It represents CD11B western blot analysis in Figure 6C.

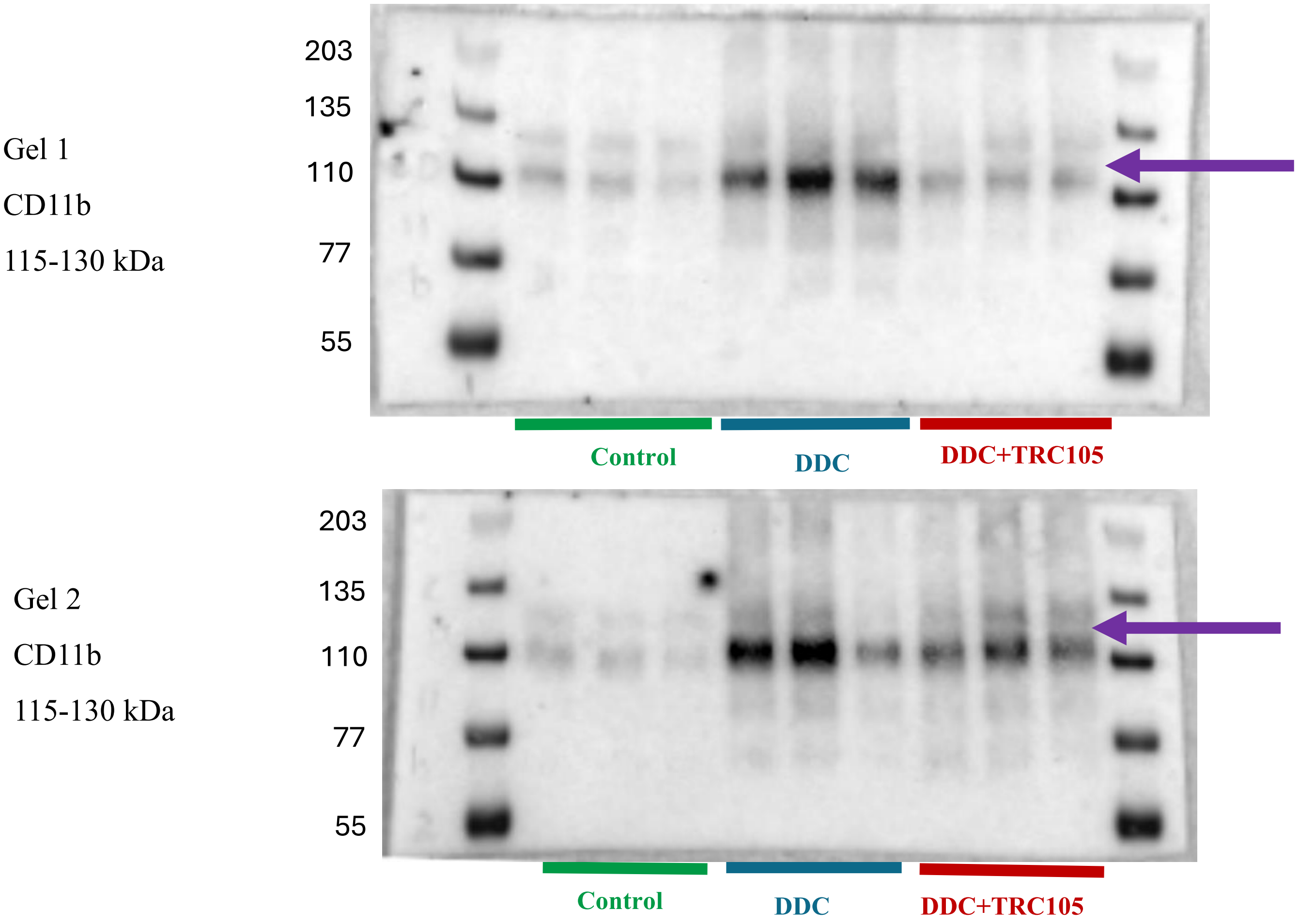

Supplement: Supplementary file 1 — Supplementary Material 1 [file 18_2026_6212_MOESM1_ESM.pdf]
