# Supplementary material for: Anti-endoglin monoclonal antibody TRC105 prevents the increase of liver inflammatory biomarkers in a mouse model of cholestasis
Source: Cell Mol Life Sci. 2026 Apr 29;83(1):255. doi: 10.1007/s00018-026-06212-2 (PMC13272742; doi:10.1007/s00018-026-06212-2)
Supplement: Supplementary file 2 — Supplementary Material 2 [file 18_2026_6212_MOESM2_ESM.pdf]

Table 1- Pre-designed TaqMan® Gene Expression Assay kits (Life Technologies) used for quantitative real-time RT-PCR:

Gene Symbol	Life Technologies Cat. Number/Assay number
Tgfb1	Mm01178820_m1
Acta2	Mm01546133_m1
Col1a1	Mm00801666_g1
Pdgfb	Mm00440677_m1
Eng	Mm00468256_m1
Gapdh	Mm99999915_g1
Ccl2	Mm00441242_m1
Il-1β	Mm00434228_m1
Ly6c	Mm00841873_m1
Tnf-α	Mm00443258_m1
