# Supplementary material for: Anti-endoglin monoclonal antibody TRC105 prevents the increase of liver inflammatory biomarkers in a mouse model of cholestasis
Source: Cell Mol Life Sci. 2026 Apr 29;83(1):255. doi: 10.1007/s00018-026-06212-2 (PMC13272742; doi:10.1007/s00018-026-06212-2)
Supplement: Supplementary file 3 — Supplementary Material 3 [file 18_2026_6212_MOESM3_ESM.pdf]

Table 2- Primary and secondary antibodies used in the Western blot:

Primary antibody	Source	Dilution	Secondary antibody	Dilution
**ENG**	Abcam (ab221675)	1:1000	Anti_Rabbit	1:2000
**p-SMAD1/5/9**	Biosciences (31-1379-00)	1:500	Anti_Rabbit	1:2000
**ID1**	Abcam (ab134163)	1:1000	Anti_Rabbit	1:1500
**p-SMAD2/3**	Abcam (ab276140)	1:500	Anti_Rabbit	1:2000
**PAI-1**	Abcam (ab222754)	1:500	Anti_Rabbit	1:1500
**MMP-14**	Abcam (ab51074)	1:2000	Anti_Rabbit	1:4000
**sENG**	Abcam (ab221675)	1:800	Anti_Rabbit	1:1000
**p65 NF-κB**	Abcam (ab16502)	1:500	Anti_Rabbit	1:1500
**ICAM-1**	R&D Systems (AF796)	1:1700	Anti_Goat	1:6000
**VCAM-1**	Cell Signaling (#32653)	1:1000	Anti_Rabbit	1:2000
**Galectin-3**	Invitrogen (14-5301-82)	1:1000	Anti_Rat	1:2000
**ET-1**	Abcam (ab216598)	1:400	Anti_Rabbit	1:1000
**p-eNOS**	Santa Cruz Biotechnology (sc-21871-R)	1:500	Anti_Rabbit	1:2000
**eNOS**	Santa Cruz Biotechnology (sc-654)	1:200	Anti_Rabbit	1:2000
**CD11b**	Abcam (ab75476)	1:700	Anti_Rabbit	1:3000
**GAPDH**	Cell Signaling (#2118)	1:8000	Anti_Rabbit	1:10000
