# Supplementary material for: Anti-endoglin monoclonal antibody TRC105 prevents the increase of liver inflammatory biomarkers in a mouse model of cholestasis
Source: Cell Mol Life Sci. 2026 Apr 29;83(1):255. doi: 10.1007/s00018-026-06212-2 (PMC13272742; doi:10.1007/s00018-026-06212-2)
Supplement: Supplementary file 4 — Supplementary Material 4 [file 18_2026_6212_MOESM4_ESM.pdf]

Table 3- Primary and secondary antibody used in the immunohistochemistry:

Primary antibody	Source	Dilution	Secondary antibody	Dilution
**Galectin-3**	Invitrogen (14-5301-82)	1:250	Anti_Rabbit	1:200
